# AI algorithms and IoT platforms for anomaly and failure prediction in industrial machinery—systematic review

**DOI:** 10.3389/frai.2026.1799522

**Published:** 2026-03-26

**Authors:** Mario Esteban Marín Vásquez, Juan Carlos Blandón Andrade, Alonso Toro Lazo, Jesús Alfonso López Sotelo

**Affiliations:** 1 Systems and Telecommunications Engineering Program, Catholic University of Pereira, Pereira, Colombia; 2 Faculty of Engineering and Basic Sciences, Autonomous University of the West, Cali, Colombia

**Keywords:** anomaly analysis, anomaly detection, artificial intelligence algorithms, failure classification, failure prediction, IoT, software platform

## Abstract

Predictive Maintenance (PdM) focuses on anticipating potential failures in industrial machines by the monitoring key parameters. Artificial Intelligence (AI) provides algorithms that can be used for this purpose. Specialized literature mentions that some companies need to adopt more proactive and predictive strategies in managing of industrial maintenance. This study aims to conduct a Systematic Literature Review (SLR) on Artificial Intelligence algorithms and software and IoT platforms used for anomaly and failure prediction. The method includes of six main phases: (i) defining the research questions; (ii) conducting a search process; (iii) establishing exclusion and inclusion criteria; (iv) performing a quality assessment of studies; (v) collecting data; and (vi) analyzing the data. The findings show that the main AI techniques for PdM are classified as: (i) machine Learning-based methods; (ii) neural networks-based methods; and (iii) knowledge transfer-based methods. Nine software and IoT technologies were identified to support maintenance operations. Additionally, it is discussed how Machine Learning and Deep Learning algorithms perform well in fault classification, prediction, Remaining Useful Life (RUL) estimation, and diagnostic tasks. They can also be applied in earlier stages, such as data preprocessing and feature extraction. Finally, it is shown that knowledge transfer can improve AI algorithms when sudden changes occur in data and their relationships. In conclusion, the AI technologies identified can significantly contribute to predicting failures in industrial machinery.

## 1 Introduction

With the emergence of new technologies related to Industry 4.0, Predictive Maintenance (PdM) has become increasingly important (Cotrino Herrera et al., 2025). This machinery method mainly focuses on monitoring machinery condition to prevent failures that could lead to high costs and performing maintenance only when necessary, utilizing the latest Industry 4.0 technologies (Craveiro et al., 2019), such as IoT (Atzori et al., 2010), AI (Luger, 2005), Big Data (Moradi et al., 2019), and Cloud Computing (Yang, 2025), among other aspects. One of the main advantages of PdM is cost savings on maintenance, with estimated reductions ranging from 25% to 35%, as well as a decrease in failures, with an elimination rate between 70% and 75%. Additionally, there is a 35% to 45% reduction in asset downtime, a 25% to 35% increase in production, and improvements in the safety of personnel, the company, and the environment (USDOE et al., 2010; Gao et al., 2015; Lazzaro et al., 2024).

According to Lazzaro et al. (2024), corrective maintenance is the oldest and most common maintenance strategy. It involves performing maintenance and repairs after a failure has already occurred. As a result, industrial assets often experience recurring and significant failures that impact not only the specific machine but also the entire production system, leading to unplanned activities that, according to Ragnoli et al. (2024), include part replacements, major production losses due to machine shutdowns, contamination, and even serious accidents involving personnel. These situations create additional problems, such as high costs caused by production downtime for repairs, financing of related activities, and the time needed to perform maintenance (Usher et al., 1998; Löfsten, 1999; Gajdzik, 2014; Vilarinho et al., 2017).

Maintenance management has evolved alongside the development of the manufacturing industry (Atkin and Brooks, 2021; Duffuaa et al., 2001; Ahmad and Kamaruddin, 2012; Dhillon, 6 16). Because of its essential role in ensuring the efficient operation of production processes, this activity has been the focus of various methodologies and approaches over time (Mangano and De Marco, 2014; De Marco et al., 2010; Patton, 2006). As evidence, there has been a growing use of maintenance activities for real-time monitoring, forecasting, and health management aimed at failure prediction in recent years (Park et al., 2022).

This Systematic Literature Review (SLR) aims to identify the main Artificial Intelligence algorithms used for anomaly analysis and classification, failure prediction and classification, as well as the primary software and IoT platforms that support their implementation. The research questions guiding this study are: (i) *what AI algorithms can be used for anomaly analysis and failure prediction-classification?*; and (ii) *which software and IoT platforms implement AI algorithms for failure prediction-classification?*.

While previous SLRs on AI-based predictive maintenance (PdM) have mainly concentrated on general machine learning methods for fault detection and IoT-enabled condition monitoring (e.g., CNNs/LSTMs for anomaly prediction and sensor data analysis), they often miss important aspects of industrial deployment. This review fills these gaps by focusing on knowledge transfer between different PdM tasks, edge and IIoT platforms for real-time processing, and resilience against cyber-attacks in distributed settings-areas that are underrepresented in existing summaries. This study aims to be a useful resource for industrial companies dealing with maintenance challenges, guiding them to adopt more effective and beneficial approaches for their production systems, and offering new insights for developing secure and transferable PdM solutions in Industry 4.0 environments.

The structure of this article is as follows: Section 2 explains the method used in the study; Section 3 shows the results; Section 4 discusses the study's findings; and finally, Section 5 offers the conclusions.

## 2 Method

A Systematic Literature Review (SLR) identifies and analyzes relevant available information for a specific area of knowledge, aiming to provide a reliable overall view based on evidence that enables drawing solid conclusions from previous studies (Medina Otalvaro et al., 2022). The method used for this research is the one proposed by Kitchenham et al. (2009), and its phases are shown in Table 1.

**Table 1:** Stages of the method.

**Stage**	**Description**
Research Questions	The topic to be investigated is established.
Search Process	The method used for data collection is defined.
Exclusion and Inclusion Criteria	The exclusion and inclusion criteria for filtering the search process results are determined.
Quality Assessment	The relevance and correct execution of the exclusion and inclusion criteria are verified.
Data Collection	The relevant information to be extracted from each search result is defined.
Data Analysis	The information is organized and analyzed.

The research questions will guide the process and objectives of the study. During the search process, the necessary information is gathered to answer the research questions, which involve several phases: (i) defining the type of search in the databases, whether manual or automated; (ii) selecting the databases that will provide the information, ensuring they are indexed databases; (iii) designing the search equations, which include a combination of keywords, Boolean operators, and other restrictions to initially segment the information within the databases; and (iv) if the search is automated, executing the search equations in each selected database to obtain the initial results for answering the research questions (Kitchenham et al., 2009).

The inclusion and exclusion criteria serve as filters that help identify and prioritize the most relevant information aligned with the objectives of the SLR. This process occurs in three steps: (i) defining the inclusion criteria, which highlight the desirable characteristics of the studies that will be part of the final collection, ensuring consistency between the objectives and the evidence; (ii) establishing the exclusion criteria, which consist of characteristics used to determine whether a search result should be discarded and are vital for refining the selection; and (iii) choosing the relevant documents, which act as a key filter before moving on to data extraction, ensuring the study's collection aligns with the research goals (Kitchenham et al., 2009).

In the quality assessment, custom evaluation criteria tailored specifically for this SLR were utilized, based on four key aspects: (i) clarity in describing the PdM task, (ii) dataset description, (iii) clarity in defining evaluation metrics, and (iv) comparison against other methods under identical conditions. Each criterion was rated descriptively as 'fully addressed,' 'partially addressed,' or 'not addressed' by three independent reviewers, with average scores calculated across reviewers. In cases of significant disagreement (e.g., differences spanning more than one category), the reviewers engaged in dialogue to reach consensus, ensuring methodological rigor. During data collection, details are obtained that make it possible to identify each result through a table that indicates the information to be extracted from each document. In data analysis, the information is presented more effectively, and an analysis and interpretation are carried out (Kitchenham et al., 2009).

## 3 Results

### 3.1 Research questions

A total of two research questions were established, based on an initial exploratory review of predicting anomalies and failures in industrial machinery, to clearly define the scope of the analysis and focus on a specific approach. The questions are as follows:

1. What AI algorithms can be used for anomaly analysis and failure prediction-classification?
2. Which software and IoT platforms implement AI algorithms for failure prediction-classification?

### 3.2 Search process

#### 3.2.1 Definition of the search process

An automated search was chosen to gather a large number of relevant and precise results that would help achieve the objectives of the SLR.

#### 3.2.2 Selection of databases

The databases chosen were IEEE Xplore, Web of Science, Scopus, and SpringerLink, as they provide access to peer-reviewed scientific literature, ensuring the quality and reliability of the included studies.

#### 3.2.3 Search equations

Three search equations were established to address each research question. The equations are shown below:

1. ((“Failure classification”) AND (“Failure prediction”)) AND ((“Artificial Intelligence Algorithms”) OR (“Deep learning Algorithms”) OR (“Machine learning Algorithms”))
2. (“software platform” OR “software platforms”) AND (AI OR IA OR “artificial intelligence” OR “machine learning” OR “deep learning”) AND (“fault prediction” OR “fault classification” OR “fault detection” OR “failure prediction” OR “failure classification” OR “failure detection”) AND (“algorithms”)
3. (“internet of things platforms” OR “internet of things software” OR “IoT platforms” OR “IoT Software”) AND (“Failure Prediction” OR “Failure Classification” OR “Failure detection” OR “Fault Prediction” OR “Fault Classification” OR “Fault detection”) AND (“Artificial Intelligence Algorithms” OR “Machine Learning Algorithms” OR “Deep Learning Algorithms”) AND (“Industry”)

The third equation explicitly combines platform-related terms (“software platform”, “software platforms”, “internet of things platforms”, “IoT platforms”, “IoT software”) with AI and failure prediction/classification terms in industrial contexts to retrieve studies that not only propose algorithms but also report their implementation on software or IoT platforms.

#### 3.2.4 Initial search

Table 2 shows the results from applying each search equation in the selected databases before using filters. This allows us to see the total number of documents initially retrieved by each equation in each source, prior to applying the inclusion and exclusion criteria. The initial search in the selected databases was conducted from January 1 to December 31, 2024.

**Table 2:** Initial search results.

**Search equation**	**Web of science**	**Scopus**	**Springer**	**IEEE xplore**	**Total**
1	992	17	11	1,002	2,022
2	56	205	221	55	537
3	23	122	46	73	264
Total	1,071	344	278	1,130	2,823

### 3.3 Inclusion and exclusion criteria

This stage helped reduce the number of results from the initial search, allowing for a first filter that only profiled documents matching the research objectives. The applied criteria are described below.

#### 3.3.1 Definition of inclusion criteria

The inclusion criteria were: (i) The article must relate to AI algorithms for anomaly detection or failure prediction-classification, software platforms, or IoT platforms implementing these algorithms; (ii) It should be a scientific article from a reputable database; (iii) The language must be either Spanish or English; and (iv) The article should be focused on industrial manufacturing machinery or present a case study in this field.

#### 3.3.2 Definition of exclusion criteria

The exclusion criteria were: (i) The article is incomplete; (ii) Article type (includes only peer-reviewed journal articles with primary empirical results; excludes reviews, book chapters, and conference proceedings); (iii) The article is repeated; and (iv) The publication date is earlier than January 1, 2019. This temporal restriction was applied to focus on contemporary PdM approaches driven by recent advances in AI, IoT, and computational infrastructures. We acknowledge this time window as a limitation and clarify that the objective of the review is to examine the state of the art in recent methodologies, rather than to provide a historical overview of the field.

#### 3.3.3 Selection of relevant documents

For selecting documents, the review focused on the title, abstract, keywords, conclusions, and finally, the introduction, in that order. If doubts about relevance remained after this process, the full document was read to make a final decision. It is also important to note that relevance was based on whether the study was highly related to industrial manufacturing machinery. The number of results per search equation in each database is shown in Table 3.

**Table 3:** Selection of relevant documents.

**Search equation**	**Web of science**	**Scopus**	**Springer**	**IEEE xplore**	**Total**
1	1	1	1	6	9
2	5	1	2	1	9
3	5	5	1	3	14
Total	11	7	4	10	32

#### 3.3.4 Flow chart of screening and filtering

**Figure 1:**
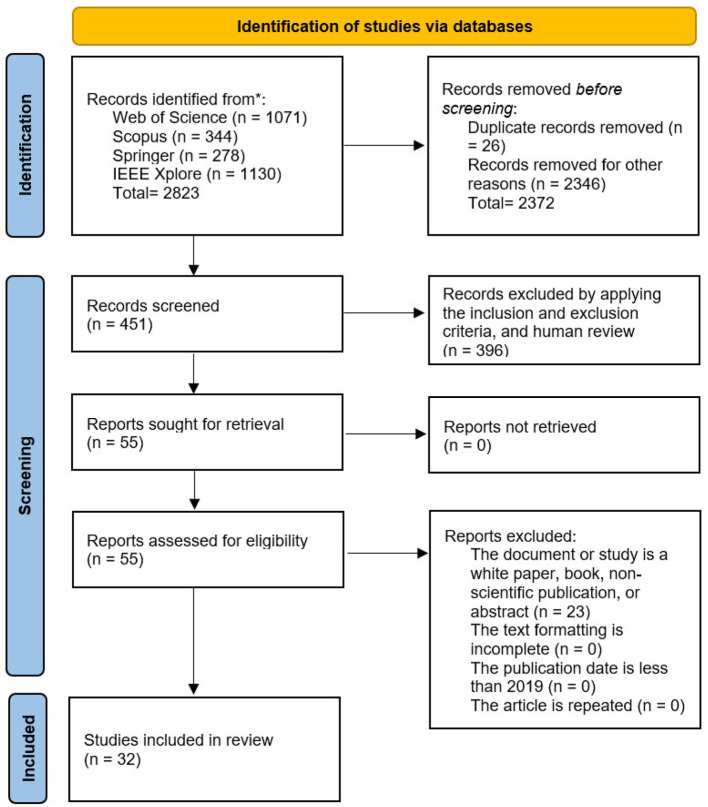
PRISMA-statement-based screening and filtering flow chart.

Figure 1 shows a PRISMA-based flowchart that illustrates the screening and filtering process. It lists the four databases from which articles were retrieved, the criteria used to exclude ineligible records, and the final group of studies included.

In the identification stage, 2,823 records were collected from four databases: Web of Science (1,071), Scopus (344), Springer (278), and IEEE Xplore Digital Library (1,130). Before screening, 2,372 records were removed (26 duplicates and 2346 records excluded for various reasons, including filtering). During the screening process, 451 records were reviewed, and 396 were excluded by applying the inclusion and exclusion criteria, and human assessment because their results were unclear or the technologies used were not relevant. This left 55 reports for full-text retrieval. In the eligibility phase, these 55 studies were evaluated, and 23 documents were excluded because they were white papers, books, or not scientific publications. Consequently, 32 studies met the inclusion criteria and were included in the review.

### 3.4 Quality assessment

To ensure that the selected documents meet quality standards, the method (Kitchenham et al., 2009) allows multiple researchers or reviewers to discuss any significant differences of opinion regarding the application of an inclusion or exclusion criterion to a particular document. Therefore, three independent reviewers used an explicit checklist of key criteria: (i) clarity in describing the predictive maintenance (PdM) task; (ii) minimal description of the dataset; (iii) clarity in defining evaluation metrics; and (iv) comparison of their proposal against other methods under the same conditions. Each criterion was rated descriptively as “fully addressed,” “partially addressed,” or “not addressed,” and average scores across reviewers were calculated for each criterion. In cases of significant disagreement (e.g., differences in more than one category), the reviewers discussed their findings to reach a consensus, thus ensuring methodological rigor.

### 3.5 Data collection

At this stage, a structured form was created to gather all the relevant information needed to answer the research questions. The form was reviewed and approved by all team members to ensure the fields were relevant and sufficient. It included key variables required for the next phase of analysis, as well as data that allowed for the identification of individual documents. The form used is shown in Table 4.

**Table 4:** Data collection form.

**Field**	**Description**
Títle	The title of the study.
Authors	The authors of the study.
Topic	The central focus of the analyzed study, that is, the specific topic or research problem that the article seeks to address.
Dataset	The size of the dataset used.
Variables	The input elements or factors measured and analyzed within the reviewed study.
Algorithm	The AI method proposed by the study for anomaly analysis or failure classification-prediction.
Platforms	The software or IoT platforms used for failure classification-prediction.
Evaluation criteria	The main metrics by which the authors evaluated the proposed method.

### 3.6 Data analysis

This section presents all the information extracted from the articles using a form designed to directly address the research questions, primarily focusing on artificial intelligence algorithms and the software and IoT platforms used for anomaly analysis, classification, or failure prediction in industrial manufacturing machinery. In this study, no quantitative meta-analysis was conducted; instead, a narrative synthesis was performed due to the significant heterogeneity of predictive maintenance tasks, datasets, and evaluation metrics among the included studies. The main similarities among the studies are identified to group the findings into thematic categories, making it easier to interpret the results. The studies were not grouped by task (RUL, fault classification, anomaly detection, etc.) or data type, since many of the analyzed studies performed different tasks and used different datasets. Therefore, the reported performance metrics derive from diverse datasets and tasks specific to each study, so they should not be compared numerically but instead evaluated based on their performance in each specific task. For this reason, the main findings are summarized in Supplementary Table S1, which group algorithms, tasks, and reported metrics to provide a more synthetic, comparative view of the studies rather than a simple author-by-author listing.

#### 3.6.1 AI algorithms for anomaly analysis and failure prediction-classification

Data preprocessing involves converting data into a format suitable for AI models, including techniques like normalization and cleaning. Feature processing, on the other hand, focuses on extracting relevant information, selecting representative subsets, and reducing data size through feature combination or removal, thereby providing useful data for training (Ragnoli et al., 2024).

### 3.7 Techniques based on machine learning

Machine Learning involves using models and algorithms to analyze available data with mathematical and statistical tools, to recognize patterns and produce reliable predictions about situations not directly observed (Akyaz and Engın, 2024). Support Vector Machine (SVM) is a type of kernel-based method used for data prediction through classification and regression, including fault diagnosis. It aims to find a boundary line—called a hyperplane—that maximizes the distance from the data points to reduce errors and separate the data into categories (Akyaz and Engın, 2024; Liu et al., 2022; Kazemi et al., 2021).

(Akyaz and Engın 2024) developed a predictive maintenance system with an IoT architecture that captures vibration, temperature, and current signals from sensors to predict failures in Bulk Continuous Filament (BCF) machines used in artificial yarn production. The system has five stages: data acquisition, preprocessing, feature selection, model training, and system deployment. For training, tests were conducted using four machine learning algorithms—Random Forest (RF), Support Vector Regression (SVR), Decision Tree (DT), and Gaussian Process Regression (GPR)—along with one deep learning algorithm, a Deep Neural Network (DNN). The results showed that the DNN achieved the highest accuracy at 96%, but the SVM was considered more suitable due to its better balance between precision, computational resources, and speed.

Kazemi et al. (2021) proposed a hybrid approach consisting of an Extended Kalman Filter (EKF) and three Support Vector Machines (SVM) for fault detection and classification in three-phase power transformers. The EKF was used to estimate current values in the primary and secondary windings and identify faults through differences in current, while the three SVMs performed different tasks: the first determined the type of fault among five classes, the second identified the fault location within two categories, and the third classified fault severity across three levels. This method achieved an overall accuracy between 98% and 99%, demonstrating high robustness to noise and measurement errors, and outperforming the algorithms it was compared to.

Yan et al. (2024) proposed a method for anomaly detection and Remaining Useful Life (RUL) prediction of bearings based on a dual-bound SVDD with dynamic update (DDSVDD). The dynamic dual-bound model introduced a relaxation strategy to improve the selection of support vectors and reduce false positives, enabling it to distinguish samples close to the hypersphere boundary. Furthermore, the Otsu method was used to dynamically adjust the dual boundaries of the model, with spatial and temporal correlations between local-global memory representations through GRU cells, accounting for the evolution of the bearing life cycle, where displacement of the hypersphere center allows for machinery health assessment and end-of-life monitoring. In the conducted tests, the model performed well, showing an average F-SCORE of 97.03%, an accuracy of 96.4%, and a training time of 3.4 minutes.

In the case of Thoppil et al. (2022), a model was developed for predictive maintenance of CNC spindle bearings, specifically to estimate Remaining Useful Life (RUL) from vibration signals. The proposed model included a feature selection process using Neighborhood Component Analysis (NCA) in the time, frequency, and time-frequency domains. Subsequently, hyperparameter tuning was performed through Bayesian optimization, using repetition and mean square error (MSE) calculations to select the best SVM configuration and minimize the difference between actual and predicted RUL. From the tests conducted, it was observed that the proposed NCA-Bayesian optimized SVM model achieved a Root Mean Square Error (RMSE) of 206.23, which is considered acceptable for predictive maintenance applications in industrial environments.

Finally, Zhang et al. (2021) developed a system for fault detection and classification in rotating machinery through vibration monitoring. They used a model called Feature Oriented Support Vector Machine (FO-SVM) that optimizes feature selection, integrated into an IoT setup with Industrial Wireless Sensor Networks (IWSNs) and the ThingSpeak platform. The FO-SVM model employs statistical learning before fault classification to select the most relevant features, considering a broad range of machine parameters and different fault scenarios. The model attained an accuracy of 98.2% in real-time fault classification in the cloud.

**Decision trees** are an inductive, supervised learning algorithm that recursively divides input data through attributes, assigning scores or labels to nodes, and using decision rules to predict outcomes, which can be intuitively interpreted thanks to their visually tree-like structure (Park et al., 2022; Liu et al., 2022; Tran et al., 2021). One of the algorithms based on Decision Trees is Random Forest, which trains multiple trees using sample dimensions and features, in addition to employing strategies such as bagging and voting to reduce the risk of overfitting and improve the model's generalization capability (Akyaz and Engın, 2024; Tran et al., 2021).

Park et al. (2022) presented an automatic labeling method using semi-weakly supervised learning in predictive maintenance applications for bearings. This approach takes the vibration signal, transforms it using FFT, and extracts a set of 130 statistical features. These features were used as input for a labeling model trained with a small labeled dataset, which then generated labeling functions through Snorkel, creating automatic labels for unlabeled data. These functions served as training input for a deep neural network (DNN) classification model. Comparisons showed that the proposed method outperformed DNN models without automatic labeling, achieving 99% accuracy, 99% precision, 1.00 recall, 99% F1-score, and a standard deviation (std) of 0.003.

Liu et al. (2022) analyzed the relative importance of feature extraction and classification algorithms in fault diagnosis based on vibration signals. Different approaches were compared: statistical features, parametric models based on Gaussian functions, and combined features using feature stacking with various classification algorithms. The results showed that effective feature extraction has a greater impact on precision than the chosen classification algorithm. The best-performing methods were Stacking features with Decision Tree (using 7-term Gaussian) and Stacking features with BP neural network (using 5-term Gaussian), achieving an accuracy of 99.94%.

Tran et al. (2021) developed a fault diagnosis system for induction motors based on IoT and automatic learning for real-time monitoring in industrial environments. It focuses on detecting bearing failures and cyberattacks through the use of a Random Forest model within an IoT infrastructure. The proposed Random Forest achieved a total accuracy of 99.03% and an ROC-AUC of 1.0, outperforming the XGBoost and Decision Tree algorithms to which it was compared.

Tasci et al. (2023) proposed a predictive maintenance system using Machine Learning algorithms for Remaining Useful Life (RUL) prediction in the manufacturing field, based on IoT sensors integrated into a real factory, which provided data such as weight, velocity, temperature, current, vacuum, and air pressure. For this, they chose a hybrid approach, performing noise filtering, using autoencoders for feature processing, and clustering data through k-means. The results of these stages were used to train Machine Learning algorithms for failure prediction, where Random Forest (RF) and XGBoost (XGB) achieved the best performance, with the former successfully preventing approximately 42% of failures in real production lines.

Ali et al. (2023) developed a method for fault detection and classification in power transformers, based on an Ensemble Machine Learning strategy called Gradient Tree Boosting (GTB). It is supported by dissolved gas analysis (DGA) and integrated into an Edge Computing architecture using the Contact Elements for IoT platform. The GTB method works by combining various weak classifiers, such as decision trees, and uses a feature engineering strategy based on gas concentration and their relationships, while also incorporating defense strategies against adversarial attacks. Several tests were conducted by subjecting the model to perturbations to measure its robustness, including the FGSM method and the ZOO adversarial optimization attack. As a result, the proposed model achieved a testing accuracy of 89.7%, demonstrating strong performance against adversarial attacks and outperforming the other compared methods.

Ragnoli et al. (2024) developed a condition monitoring and fault prevention system focused on a multipurpose CNC machine, integrating a digital twin and a fault classification method based on regression trees (CART). The methodology includes feature extraction using Fast Fourier Transform (FFT) in the frequency domain from vibrations captured through accelerometers, which were then compressed using an Empirical Moving Average (EMA) technique. The proposed CART model allows classifying the machine state into three distinct categories: no damage, slight damage, and severe damage, by training a regression tree for each class and combining them to obtain the corresponding category. Tests were conducted comparing the model with other algorithms such as K-NN, SVM, and MLP, where the proposed method achieved the best results with a weighted accuracy of 98.7%.

In addition, other Machine Learning algorithms used for anomaly analysis, as well as fault classification and prediction, were identified in the reviewed studies, highlighting the great diversity of methodological approaches. Ghasemkhani et al. (2023) developed a method based on the K-Star classification algorithm, which they called Balanced K-Star, as it addresses the problem of data imbalance in predictive maintenance datasets for IoT-based manufacturing, using an explainable learning approach for fault prediction. This algorithm applies Bayes' theorem to select representative observations and uses undersampling to balance the data, ensuring that data reduction does not eliminate relevant examples. The goal is to subsequently use the K-Star algorithm to classify between normal and faulty conditions, while explaining the result of each fault type through a logical fault tree, thereby making the process more transparent. This method achieved the highest accuracy value (98.75%) compared to the conventional K-Star and the average of state-of-the-art models used for comparison.

Xiao et al. (2023) developed a condition monitoring system based on edge computing for process manufacturing. The solution integrates the online development of monitoring algorithms, executable program generation, and their implementation on edge nodes. Its applicability was demonstrated with a real case in aluminum cold rolling, where the proposed algorithm, called Volterra Polynomial Basis Function (VPBF), was used to predict energy consumption and oil temperature. These variables, being critical for equipment operation, were monitored by comparing their values with predefined normal ranges, serving as the basis for alerts. The algorithm showed better performance than all others with which it was compared, having lower Mean Square Error (MSE) and Mean Absolute Percentage Error (MAPE) values, and higher R^2^ coefficients, both in predicting energy consumption and oil temperature.

Han et al. (2021) proposed a feature extraction method for bearing fault signals, combining Local Mean Decomposition (LMD) and Multi-Scale Symbolic Dynamic Entropy (MSDE), along with a noise filter based on RLS. This combination was further enhanced by an Affinity Propagation (AP) clustering procedure for automatic fault type and level classification. Experiments showed that the proposed method achieved an overall accuracy of 99.38%.

Finally, Li and Zhao (2022) proposed a method for bearing degradation prediction, which improved the PSO algorithm by adding an inertia weight, a linear learning factor, and a perturbation term, thus optimizing the penalty coefficient and the kernel parameters of KELM. Consequently, they proposed WCDPSO-KELM, also testing various feature extraction methods in the time-frequency domain, such as Wavelet and Ensemble Empirical Mode Decomposition (EEMD). Experimental results showed that the latter achieved the best performance, with an average MAPE of 0.0686 and a standard deviation of 0.0013.

### 3.8 Techniques based on neural networks

A neural network is a computational model capable of learning through the backpropagation (BP) algorithm and is usually composed of an input layer, an output layer, and at least one hidden layer. Each layer contains nodes that are interconnected with adjacent layers, seeking to adjust the weights and connections so that the obtained outputs are as close as possible to the desired values (Liu et al., 2022; Verma et al., 2022).

Raja et al. (2022) developed a method based on current signal spectrum analysis for predictive maintenance in induction motor machines. The method, named Single Spectrum Based Approach, performs feature extraction using Fast Fourier Transform (FFT), distinguishing between healthy and faulty motor conditions. The extracted parameters are standardized and used to train various AI algorithms. As a result, the neural networks achieved the best performance, with accuracies between 99.1% and 99.6%, with the Wide Neural Network showing the highest performance.

Verma et al. (2022) developed a Fault Signature Analyzer (FSA) for real-time monitoring of the condition of industrial machines. This system focuses on early fault detection in the stator, using motor current analysis in the time and frequency domains, along with Wavelet Transform and AI techniques. The FSA model employs Discrete Wavelet Transform (DWT) to extract statistical features, followed by a C4.5 decision tree to select the most relevant ones. This FSA was used as input for various AI algorithms, finding that a neural network with configuration 2 achieved the best performance, with 98% accuracy, surpassing other FSA algorithms and state-of-the-art methods. Furthermore, it was observed that FSA enhances the performance of all algorithms with which it was integrated.

(Yotov and Aleksieva-Petrova 2024) proposed a data-driven prediction method for analyzing sensor signals in manufacturing processes, with the main objective of predicting tool wear in shear cutting processes, particularly the wear of the punch tool in a press. The approach combines feature extraction techniques such as TS-FRESH and TSFEL with transformations like PCA, Fourier, and Wavelet, depending on the type of algorithm used, whether shallow or deep learning. It was found that the Wavelet-CNN combination achieved the most promising results, with an accuracy of 92.3% on the first dataset and 62.4% on the second, which was considered more complex. The Wavelet-CNN architecture demonstrated high learning capacity, although with greater computational requirements.

Magadán et al. (2023) developed a fault diagnosis and Remaining Useful Life (RUL) prediction technique for bearing in electronic motors, combining data preprocessing through the Hilbert-Huang Transform (HHT) in the frequency domain with stacked autoencoders (SAE) to extract vibration features with input from a BiLSTM neural network. The objective was to learn different working conditions with a high level of robustness, accuracy, and prediction speed, achieving the best results in precision compared to other studies used for comparative purposes.

Kizito et al. (2021) developed a stylized LSTM model, which they used for various activities related to Predictive Maintenance (PdM), such as fault detection, Remaining Useful Life (RUL) prediction, and failure probability estimation within a time window in electric motors. The model was compared with Random Forest, where the stylized LSTM achieved better accuracy (93.4%) and precision (100%) in fault prediction, as well as lower MAE (0.075) and MSE (0.008) in RUL prediction. Both models achieved a coefficient of determination (R^2^) of 92%. Moreover, the stylized LSTM could predict the occurrence of a fault within a 24-hour period before it actually happened.

Chen et al. (2023) presented a method based on Capsule Networks (CapsNet) enhanced with dynamic pruning and multiscale mutual information maximization for the diagnosis of compound faults in rotating machinery, called DPMI-CapsNet. Compared with other methods, it demonstrated excellent performance, achieving an average accuracy of 99%.

On the other hand, Lazzaro et al. (2024) built a Predictive Maintenance (PdM) system through cutting force sensor monitoring in a lathe machine, integrating technologies such as Cyber-Physical Systems (CPS) and Edge Computing to reduce latency in cloud-based predictive maintenance systems. The main goal of this continuous monitoring system was to use influencing parameters in chip formation during the turning process to diagnose anomalous patterns that deviate from the characteristics defined by ISO 3685, thus preventing process interruptions that could cause a fault. To achieve this goal, experiments were carried out with different types of AI algorithms, where the neural networks achieved accuracies between 96% and 98%, with Bilayered NN and Medium NN obtaining the best performance, reaching 98.1% accuracy.

Tran et al. (2023) proposed a Fault Recognition and Correction (FRC) scheme based on IoT and Deep Learning for induction motors, using vibration signals to extract bearing fault features. The AI algorithm implemented was a Deep Neural Network (DNN), which was tested through various experiments, including Fault Data Injection (FDI) to assess model robustness and comparisons with other AI algorithms. The proposed DNN achieved the best performance, with an accuracy of 99.84%, demonstrating its effectiveness and reliability in industrial environments with cybersecurity threats.

De Vita et al. (2020) developed a complete hardware and software infrastructure for data collection, management, and analysis from multiple sensors in industrial environments, using both Cloud Computing and Edge Computing. The sensors provided acoustic, distance, current, and temperature signals, designed for a wide range of industrial applications. The system was developed to detect anomalies and predict failures through data fusion from multiple sensors and multilayer learning, turning failure prediction into a multiclass classification problem. Before the DNN processed the data, preprocessing was performed to eliminate atypical values, transforming the signal into the frequency domain using FFT and employing time-displacement features. Finally, the DNN consisted of two main layers: feature extraction and classification. The method was tested using a full-scale replica of an industrial plant, where it was observed that the sensor data fusion approach improved the performance of the DNN compared with a monolithic DNN and an SVM.

Jiang et al. (2024) proposed a real-time health monitoring platform for industrial robots, using cloud computing and edge computing, along with deep learning models and time series analysis. Their goal was to provide a low-code solution capable of performing anomaly detection and failure prediction tasks. To this end, they implemented two deep learning models on their platform: AE-DTW, which uses autoencoders to modify the loss function to diagnose sudden failures, and Transformer-SD, which improves the transformer model through signal decomposition, allowing it to learn long-term patterns and predict failures more effectively. The proposed approach was tested on datasets collected over two years, containing more than 100 million data points from the time series of 13 industrial robots, achieving an F1-score of 84% for AE-DTW and 77% for Transformer-SD in their respective tasks.

Maguluri et al. (2024) developed a framework for AI-assisted predictive maintenance in hybrid roll-to-roll (R2R) manufacturing processes, combining a multisensor IoT infrastructure–which collects variables such as temperature, vibration, pressure, acoustics, and vision–with a model called Contrastive Predictive Coding (CPC) to improve real-time anomaly detection and fault prediction without relying on large labeled datasets. First, a data processing pipeline is executed, consisting of four steps: cleaning, normalization, resampling, and feature extraction based on domain knowledge and statistical analysis. Then, the CPC model processes the data through its encoder network (a CNN or LSTM) and context network (an RNN), and it is trained using a contrastive loss function. This model was used as input for other AI algorithms for anomaly detection and fault prediction. In the study, a neural network (NN) was employed for testing, and results were compared with those obtained using a Random Forest (RF) and a neural network without CPC. Significant improvements were observed in metrics such as AUC-ROC (98%), accuracy (95.2%), and F1-score (93%), in addition to generating alerts up to 20 minutes before an actual failure occurred. This demonstrates that contrastive self-supervised learning can extract useful features that substantially improve predictive maintenance processes in industrial environments.

Finally, Niu et al. (2023) developed a method called SR-DEEP, which combines sparse representation and deep neural networks–specifically, a regression network based on a multilayer perceptron (MLP) with an adaptive strategy for regularization parameter tuning. This approach improves the precision of fault diagnosis in rotating machinery bearings by adapting these parameters. Using this method, they achieved average accuracies of 100% and 99.20% on two rotating machinery datasets, outperforming other compared methods.

### 3.9 Techniques based on knowledge transfer

Knowledge transfer is a method that seeks to improve the performance of new tasks based on previous experiences (Liu et al., 2024; Mao et al., 2023; Zhao et al., 2022; Cao et al., 2024). Within this approach, transfer learning uses existing knowledge to acquire new knowledge by identifying similarities between the existing knowledge (source domain) and the new knowledge to be learned (target domain) (Liu et al., 2024).

There is also domain adaptation and its adversarial approach, which consists of finding a shared feature space between the source and target domains, using adversarial training strategies, the Maximum Mean Discrepancy (MMD) metric, among others (Mao et al., 2023). Finally, transfer learning aims to transfer knowledge from large models to smaller models, so that the smaller model achieves similar performance with fewer parameters (Cao et al., 2024).

Liu et al. (2024) developed a rotating machinery fault prediction model based on vibration and acceleration signals, combining **transfer learning** and deep learning to address generalization issues in model recognition and feature extraction. The proposed model, called Deep Adaptation Residual Network (DARN), operates by constructing a time-frequency spectrum, followed using a pretrained residual neural network (ResNet-50) for feature extraction. Different layers were later connected for prediction (classification, transfer, and central), minimizing the effects of transfer bias. Multiple tests showed that the model achieved superior performance in rotating machinery fault diagnosis, with an average accuracy of 99%.

Zhao et al. (2022) proposed a method called Joint Adversarial Domain Adaptation (JADA), used for bearing fault diagnosis under different operating conditions. This method seeks to reduce the difference between the distribution of training data (source domain) and test data (target domain) through adversarial domain adaptation. The model generates a feature representation using a Convolutional Neural Network (CNN), which is then processed by a classifier composed of three fully connected layers and an adversarial discriminator. The method achieved highly accurate classification results and strong domain adaptation capability, reaching an average accuracy of 99.51% using the DDS dataset and 99.67% with the CWRU dataset, outperforming all comparative methods.

Mao et al. (2023) developed a Remaining Useful Life (RUL) prediction model designed for unknown bearing operating conditions. This model, called Self-Supervised Deep Domain Adversarial Regression Adaptation, is based on self-supervised deep learning with adversarial domain adaptation. It enables **knowledge transfer** from historical data in limited-data environments. The model consists of a TL-TSRF module, which uses a Tensor-LSTM to recursively predict the degradation sequence of bearing life based on data outside the line, generating pseudo-labels for RUL in the target domain. Subsequently, a CNN is used for the extraction of the most relevant features, adapting the process to a regression problem.

Cao et al. (2024) created a framework called Online Knowledge Distillation (OKD), which applies online knowledge distillation for machinery health prognosis, focusing specifically on predicting the remaining time before a failure occurs, using edge computing. This method trains two neural networks simultaneously: the Teacher Network, which is more complex, and the Student Network, which is lighter and more suitable for resource-limited devices. Both networks interact so that the first transfers knowledge to the second. In the case study, the Teacher Network was a Complex Domain Extension Network (CDEN) and the Student Network was a standard CNN. OKD showed improvements in metrics such as RMS, SMAPE, and Score compared to the vanilla CNN without distillation and other state-of-the-art algorithms, demonstrating its effectiveness in scenarios with computational constraints.

#### 3.9.1 Software and IoT platforms implementing ai algorithms for fault prediction-classification

**Matlab** is a platform equipped with tools for the development and training of Machine Learning models, such as the Machine Learning Toolbox, which includes applications like Classification Learner and Regression Learner. In addition, integration with Simulink allows for system implementation and simulation, further expanding its capabilities (Lazzaro et al., 2024; Kazemi et al., 2021; Xiao et al., 2023). Thoppil et al. (2022) developed a model for predicting the Remaining Useful Life (RUL) of CNC spindle bearings, using Neighborhood Component Analysis (NCA) together with a Bayesian Optimized Support Vector Machine (SVM), achieving an RMSE of 206.23. Kazemi et al. (2021) used Matlab's Classification Learner for training and validating three SVM classifiers, designed to determine the type, location, and severity of faults, respectively, detected by the EKF. This implementation was integrated with the PSCAD/EMTDC platform, used for simulating faults in three-phase power transformers, thus generating the data for training and testing. Subsequently, dSPACE1104 was employed for Hardware-in-the-Loop (HIL) testing of the implemented EKF, which serves as input for the SVMs in Matlab's Classification Learner.

Lazzaro et al. (2024) created a process monitoring system for turning operations using IoT technologies, such as Cyber-Physical Systems (CPS), force sensors, and Edge Computing, with the aim of reducing latency in cloud-based predictive maintenance systems. To this end, it was necessary to detect chip formation patterns during the turning process and, using ISO 3685 as a reference, identify anomalies in the same through various influencing parameters. To detect these anomalies, experiments were conducted to determine which AI algorithm was most suitable for the task, using the Classification Learner App included in Matlab R2021's Statistics and Machine Learning Toolbox. This software allows training both supervised and semi-supervised algorithms for binary and multiclass classification. Finally, the best results were obtained by the neural networks trained with the TensorFlow library.

**ThingSpeak** is another IoT platform identified in several analyzed studies as a tool for developing IoT-based predictive maintenance systems, such as those involving sensors and real-time communication protocols. Its adoption is due to its multiple features, including the ability to manage, process, and visualize real-time data, as well as issue automatic alerts for abnormal conditions. Furthermore, it allows users to execute code directly in the cloud through integration with MATLAB, eliminating the need for dedicated servers or external software development. This functionality, combined with its middleware structure between IoT devices and the application layer, contributes to a robust environment in terms of security and privacy for modern industrial applications (Akyaz and Engın, 2024; Zhang et al., 2021).

(Zhang et al., 2021) developed a fault diagnosis system within an IoT architecture composed of industrial wireless sensor networks and the ThingSpeak platform. This platform was used for real-time data storage and analysis, also allowing device interconnection and remote monitoring. ThingSpeak was further integrated with a Python script, in which the proposed Feature Oriented Support Vector Machine (FO-SVM) algorithm was implemented for extracting the most relevant features and fault diagnosis, achieving an accuracy of 98.2%.

(Akyaz and Engın 2024) also developed a predictive maintenance system using an IoT architecture, which captured sensor signals for fault prediction in Bulk Continuous Filament (BCF) machines used in artificial yarn production. In this case, the ThingSpeak platform was integrated, allowing the writing and execution of Matlab code. ThingSpeak used the MQTT protocol to reduce the microcontroller's energy consumption, extending the battery life. The platform also enabled the differentiation between sensor data channels to discriminate the inputs provided by sensors such as vibration, temperature, and current, facilitating their management. In addition, data were visualized immediately in the cloud, and automatic alerts were generated for electrical or mechanical maintenance when detected values exceeded the established operational limits. Finally, Matlab's Regression Learner application was used to optimize the data, allowing the authors to detect anomalies and faults in advance, thus improving decision-making capacity before their occurrence.

**Contact Elements for IoT** has proven to be highly useful in predictive maintenance tasks, and several authors chose to employ it to optimize the operation and management of IoT architectures, providing a viable and adaptable option under diverse conditions thanks to its ability to integrate sensors, manage data flows, store information in the cloud, visualize real-time signals, and facilitate decision-making through its integration with machine learning models in industrial environments (Ali et al., 2023; Tran et al., 2023).

Ali et al. (2023) built an IoT architecture for fault diagnosis in power transformers, based on the Contact Elements for IoT platform. This architecture integrates multiple remote sensors for Dissolved Gas Analysis (DGA), employing Edge Computing with the MQTT protocol and Digital Twins. It allows storing and evaluating measurements in transformers to identify fault trends in gas concentrations and their relationships. A Gradient Tree Boosting (GTB) ensemble learning method was incorporated, achieving a testing accuracy of 89.7% and demonstrating robustness against adversarial attacks such as FGSM and Zoo. This solution aligns with Industry 4.0 trends, supporting online monitoring, remote visualization, and predictive capabilities through Artificial Intelligence. These experiments show that the edge-oriented Contact Elements for the IoT architecture not only perform conventional transformer fault diagnosis but also explicitly incorporate robustness mechanisms against adversarial perturbations at the edge layer, illustrating how cybersecurity can be embedded in PdM workflows in IIoT contexts.

Tran et al. (2023) presented a Fault Recognition and Correction (FRC) method for induction motors, based on vibration signals and a Deep Neural Network (DNN) within an IoT architecture. This proposal employed the platform for online motor condition monitoring, using a neural model based on the automatic acquisition of equipment condition readings, enabling fault visualization and improving decision-making. In this way, the FRC framework goes beyond merely acknowledging cyber risks and uses false data injection scenarios at the acquisition and edge processing stages to validate the resilience of the PdM model, detecting and correcting cyber-induced anomalies before they affect higher-level maintenance decisions.

Various studies also identified several customized **platforms developed by the authors themselves**, which were shown to contribute significantly to the results in anomaly analysis and fault classification or prediction tasks. Xiao et al. (2023) designed a platform for condition monitoring that enables the creation of algorithms and their execution at the edge, as well as real-time visualization functions and compatibility with multiple communication protocols. The algorithms are designed using block diagrams converted to JSON format, which are then sent to a server that transforms this format into an M-script, subsequently into a Simulink block diagram. Finally, they are compiled into an executable program using Simulink Coder. An automatic communication protocol was added between the executable program and the communication server. The platform integrates Matlab 2017b tools. Within this system, the VPBF algorithm was designed to predict energy consumption and oil temperature in aluminum cold-rolling processes, achieving the best performance metrics (MSE, MAPE, and R^2^) compared to other algorithms.

Ragnoli et al. (2024) developed a web platform called ASSIOMI, which integrates a digital twin for monitoring and preventing failures in a multipurpose CNC machine. It allows assigning machines and users to organizations, distinguishing between base users and administrators with different permissions. Each registered machine records state indicators and anomaly signals, which are triggered when the integrated method, called CART, detects an abnormal operating condition. In tests, the system achieved a weighted accuracy of 98.7%.

Jiang et al. (2024) developed an IoT platform called PHIR, aimed at health monitoring of industrial robots, integrating Cloud Computing and Edge Computing. PHIR is a low-code platform designed to minimize the need for programming by the user. It uses Kubernetes-managed containers to encapsulate Deep Learning algorithms and data processing models, enabling simple administration. The platform includes integrated models such as AE-DTW and Transformer-SD. Through its graphical interface, users can define workflows, after which PHIR creates operators and deploys the instance based on them. The platform was tested for over two years in a real industrial environment, connected to 13 industrial robots and handling more than 100 million time-series data points. The results demonstrated its effectiveness in anomaly detection and fault prediction, representing a key contribution to maintenance in the manufacturing industry.

Additionally, **other platforms** were identified. Yousuf et al. (2024) proposed a health monitoring system for three-phase induction motors using sensors, IoT, GSM, and primarily the IoT platform Blynk for cloud-based supervision. Although it does not integrate complex AI algorithms, it enables predictive maintenance through remote monitoring of variables such as temperature, vibration, current, voltage, and rotational speed. It allows configuring rule sets to automatically generate alerts when certain thresholds are exceeded. In testing, it detected abnormal behaviors with an accuracy of 99%, demonstrating high operational efficiency and ease of use.

De Vita et al. (2020) used the Stack4Things platform for anomaly detection and fault prediction. This OpenStack-based architecture employs Industry 4.0 technologies for managing various sensors (acoustic, distance, current, vibration, temperature), performing data fusion from multiple sources. Cloud computing was used through Grafana's web panel integration, displaying alerts generated by the DNN algorithms applied to the data fusion process. Stack4Things facilitated labeling, training, inference, and data injection in IoT models using its plugin system, leveraging edge computing.

Maguluri et al. (2024) mentioned the existence of the TIP4.0 platform, an IIoT (Industrial Internet of Things) solution for predictive maintenance that integrates AI models with real-time sensor readings, enabling tasks such as fault prediction, anomaly detection, assessment, and intervention programming. Moreover, it includes the **iSTEP** engine, capable of learning how Machine Learning models converge and dynamically adjusting real-time maintenance strategies to reduce costs and increase efficiency.

Finally, it is important to note that while 28 studies (87.5%) detailed AI algorithms for anomaly detection and failure prediction, only 9 studies (28.1%) specifically identified the software/IoT platforms implementing these algorithms, revealing a significant gap between algorithm development and practical deployment capabilities.

**Figure 2:**
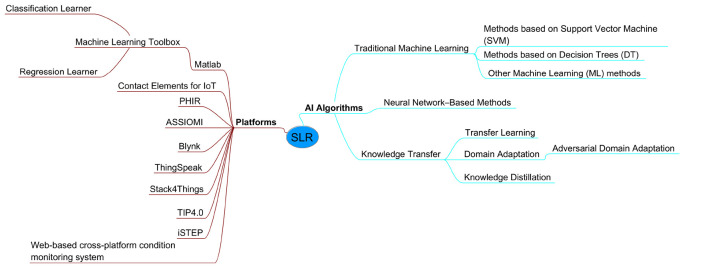
SLR summary.

Figure 2 presents a synthesis of the findings obtained throughout the Systematic Literature Review. A mind map was constructed to visually summarize the various topics relevant to the defined research questions, as well as other significant findings identified in the current literature that help complement the overall perspective.

## 4 Discussion

The purpose of this article was to conduct a SLR following the method proposed by Kitchenham et al. (2009), to identify both artificial intelligence algorithms used for anomaly analysis and fault prediction or classification, as well as the software and IoT platforms that enable the implementation of these algorithms in industrial environments. Unlike previous SLRs on AI-based PdM, which predominantly address general ML techniques for fault detection or basic IoT monitoring, this review uniquely emphasizes, based on the results obtained, knowledge transfer between heterogeneous tasks, Edge/IIoT platforms for real-time implementation, and strategies for robustness against cyberattacks in industrial environments: critical but underexplored dimensions for the scalability of Industry 4.0.

Regarding cybersecurity, only a few studies explicitly evaluate robustness against adversarial attacks or false data injection in PdM settings. In particular, the Contact Elements for IoT platform stress-tests an edge-oriented Gradient Tree Boosting model with FGSM and ZOO perturbations, while the FRC framework for induction motors validates a deep model under FDI scenarios to detect and correct cyber-induced anomalies at the edge. These examples illustrate concrete directions for integrating adversarial robustness and edge-side validation into PdM platforms, but they also highlight that systematic cybersecurity evaluation remains the exception rather than the norm in the current literature.

The results reveal a notable diversity in the approaches used by different authors, showing that research efforts have been directed in multiple directions, resulting in a wide range of technological options. These options are not mutually exclusive; on the contrary, many AI and IoT technologies demonstrate a strong capacity to complement each other.

Table 5 presents the division between the two main objectives of this SLR. It shows that numerous studies contribute simultaneously to both research questions by proposing and implementing not only AI algorithms but also software and IoT platforms that strengthen maintenance operations. Nevertheless, a significant difference was observed in the number of publications associated with each question, with a total of 31 articles focused on Artificial Intelligence algorithms, compared to only 13 studies addressing software and IoT platforms.

**Table 5:** List of articles by research question.

**Question**	**Authors**
AI algorithms for anomaly analysis and fault prediction-classification	Akyaz and Engın, 2024; Kazemi et al., 2021; Yan et al., 2024; Thoppil et al., 2022; Zhang et al., 2021; Tran et al., 2021; Tasci et al., 2023; Ali et al., 2023; Ragnoli et al., 2024; Ghasemkhani et al., 2023; Xiao et al., 2023; Li and Zhao, 2022; Magadán et al., 2023; Kizito et al., 2021; Chen et al., 2023; Lazzaro et al., 2024; Tran et al., 2023; De Vita et al., 2020; Jiang et al., 2024; Niu et al., 2023; Liu et al., 2024; Zhao et al., 2022; Mao et al., 2023; Cao et al., 2024; Park et al., 2022; Yotov and Aleksieva-Petrova, 2024; Verma et al., 2022; Liu et al., 2022; Han et al., 2021; Raja et al., 2022; Maguluri et al., 2024
Software and IoT platforms implementing AI algorithms for fault prediction-classification	Thoppil et al., 2022; Kazemi et al., 2021; Lazzaro et al., 2024; Zhang et al., 2021; Akyaz and Engın, 2024; Ali et al., 2023; Tran et al., 2023; Xiao et al., 2023; Ragnoli et al., 2024; Jiang et al., 2024; Yousuf et al., 2024; De Vita et al., 2020; Maguluri et al., 2024

This disparity is considered an opportunity for improvement that deserves greater attention, since, as evidenced in Section 3.6—Data Analysis, these platforms not only facilitate tasks related to the training, evaluation, and deployment of AI algorithms, but also support many other critical activities essential to the operation of the manufacturing industry. These include, among others real-time monitoring for determining the health status of equipment, one of the most relevant practices within the Industry 4.0 framework. This gap could be due, among other reasons, to the predominance of theoretical approaches in academia, the lack of access to real industrial platforms, or researchers' limited knowledge regarding the development and implementation of such solutions. In this context, it would be pertinent for future research to explore the causes of this disparity in depth, in order to gain the necessary knowledge to make informed decisions that encourage the adoption of software and IoT platforms in industrial environments. Table 6 shows the methods found for fault detection.

**Table 6:** Artificial intelligence methods and technologies identified in the reviewed studies.

**AI method or technology**	**Authors**
Methods based on support vector machines	Akyaz and Engın, 2024; Kazemi et al., 2021; Yan et al., 2024; Thoppil et al., 2022; Zhang et al., 2021
Methods based on decision trees	Tran et al., 2021; Tasci et al., 2023; Ali et al., 2023; Ragnoli et al., 2024; Park et al., 2022; Verma et al., 2022; Liu et al., 2022
Other machine learning methods	Ghasemkhani et al., 2023; Xiao et al., 2023; Li and Zhao, 2022; Han et al., 2021
Neural network-based methods	Magadán et al., 2023; Kizito et al., 2021; Chen et al., 2023; Lazzaro et al., 2024; Tran et al., 2023; De Vita et al., 2020; Jiang et al., 2024; Niu et al., 2023; Liu et al., 2024; Mao et al., 2023; Cao et al., 2024; Park et al., 2022; Yotov and Aleksieva-Petrova, 2024; Verma et al., 2022; Liu et al., 2022; Raja et al., 2022; Maguluri et al., 2024
Knowledge transfer-based methods	Liu et al., 2024; Zhao et al., 2022; Mao et al., 2023; Cao et al., 2024

As can be observed, several articles employed multiple categories of methods, which demonstrates once again that, depending on the specific needs of the problem, a solution can be designed based on the combination or ensemble of various types of technologies, thus achieving better results. Support Vector Machine (SVM)-based methods proved to be highly effective in various predictive maintenance applications, including fault classification and Remaining Useful Life (RUL) estimation, standing out for their high generalization capacity, robustness to noise, and precision in industrial environments (Akyaz and Engın, 2024; Kazemi et al., 2021; Yan et al., 2024; Thoppil et al., 2022; Zhang et al., 2021). Likewise, these methods, such as SVM, not only exhibited excellent performance in predictive maintenance tasks but also showed a great balance among accuracy, speed, and computational demand, making them an excellent option when a model is required to perform efficiently in industrial contexts while maintaining low resource consumption (Akyaz and Engın, 2024). Furthermore, they were capable not only of classifying faults but also of determining their location and severity level (Kazemi et al., 2021) and reducing false positives (Yan et al., 2024). Lastly, they were identified as highly versatile methods, as they can be integrated with data preprocessing, feature processing, and hyperparameter optimization techniques.

On the other hand, **Decision Tree-based methods** showed excellent results in diagnosis, classification, and fault prediction tasks, as well as in RUL estimation for industrial equipment. Random Forest, Gradient Tree Boosting (GTB), and CART exhibited accuracy values between 89.7% and 99.03%, positioning themselves as a solid option for activities related to predictive maintenance (Ragnoli et al., 2024; Tran et al., 2021; Tasci et al., 2023; Ali et al., 2023). An important point to note is that data preprocessing and feature processing techniques were once again fundamental, showing good compatibility when developed alongside other methods. Similarly, the Balanced K-Star (Ghasemkhani et al., 2023), Volterra Polynomial Basis Function (VPBF) (Xiao et al., 2023), and WCDPSO-KELM (Li and Zhao, 2022) methods were used for fault classification activities, continuous monitoring of machine conditions, and bearing degradation prediction, achieving significant performance improvements compared to the algorithms with which they were tested, outperforming them in metrics such as accuracy, coefficient of determination (R^2^), MAPE, and F-Score. These results show the growing relevance of these methods beyond traditional Machine Learning algorithms, highlighting the importance of developing new techniques in contexts where conditions are highly variable.

**Neural networks** are highly versatile, and the literature has made it clear that they can be used not only as final algorithms for anomaly analysis or fault classification/prediction, but also in earlier stages, such as data preprocessing and feature processing, preparing the input for other Artificial Intelligence algorithms discussed previously. Thanks to the considerable improvements they have undergone, neural networks tend to be more robust and better adapted to varying operating conditions, being able to process large volumes of data and model nonlinear relationships, for which they have shown promising results. They presented a wide variety of methods, such as BiLSTM (Magadán et al., 2023), stylized LSTM (Kizito et al., 2021), DPMI-CapsNet (Chen et al., 2023), Bilayered NN and Medium NN (Lazzaro et al., 2024), DNN (Tran et al., 2023; De Vita et al., 2020), AE-DTW and Transformer-SD (Jiang et al., 2024), MLP (Niu et al., 2023), among others. Despite their excellent results and performance, it should also be noted that they entail higher computational costs and, therefore, higher implementation requirements, which in some cases lead to prioritizing aspects such as training speed and computational efficiency, where other traditional Machine Learning approaches still show strong performance, though with significantly lower parameter demands (Akyaz and Engın, 2024).

Methods based on **knowledge transfer** techniques present a truly innovative and important approach, as they directly address several issues commonly encountered during the training and use of Artificial Intelligence models–particularly when there is an abrupt change in the data and their relationships (Liu et al., 2024; Mao et al., 2023; Zhao et al., 2022; Cao et al., 2024). The first key characteristic lies in the fact that knowledge transfer-based methods mitigate the impact of domain changes—that is, changes in data behavior–by searching for similarities in the feature space between the source and target domains, thereby reducing the model's dependency on a single operational environment (Liu et al., 2024; Mao et al., 2023; Zhao et al., 2022). Similarly, it is observed that training time can be reduced by leveraging prior knowledge, which is a significant advantage in industrial environments where training from scratch can often be costly and impractical (Liu et al., 2024; Cao et al., 2024). Additionally, through knowledge distillation (Cao et al., 2024), it is possible to transfer the knowledge acquired by more complex models to simpler and more efficient models, combining two major advantages: first, by transferring the knowledge from the more complex model (teacher), the performance of the latter is largely retained in the simpler model (student); and second, the simpler model becomes computationally more efficient, requiring fewer parameters than the teacher while achieving similar performance. These methods employed neural networks with excellent performance, outperforming the algorithms they were compared with, and in some cases reaching accuracy values above 99%.

**Three key aspects** were also identified that may be highly useful in the context of predictive maintenance. The first of these is **Edge Computing** which consists of running Artificial Intelligence models as close as possible to the industrial equipment–i.e., directly on the embedded boards or devices installed in the machinery for continuous monitoring (Lazzaro et al., 2024; Ali et al., 2023; Xiao et al., 2023; De Vita et al., 2020; Jiang et al., 2024; Cao et al., 2024). This technology offers multiple advantages, such as a significant reduction in latency by eliminating the need to send data to external servers for processing. Instead, the AI algorithm is deployed directly at the edge, allowing each newly captured data point to be processed immediately. This is particularly useful in cases where an anomaly is detected or a fault is predicted, and immediate action must be taken, since any delay could cause considerable adverse effects (Lazzaro et al., 2024; Ali et al., 2023; Xiao et al., 2023; De Vita et al., 2020; Jiang et al., 2024; Cao et al., 2024). However, there is also a limitation regarding the computational power of the boards used for data acquisition, as models requiring high computational capacity generally cannot be executed on them. To address this, knowledge distillation (belonging to the knowledge transfer approach) is introduced, which allows a substantial reduction in the computational resources required to execute a model, while maintaining good performance through a more complex model functioning as a teacher that transfers the learned knowledge (Cao et al., 2024). Another alternative is to use an Artificial Intelligence algorithm that does not require an amount of computational resources that the board cannot provide, such as a simple neural network or a Machine Learning algorithm based on SVM or Decision Trees.

The second approach corresponds to **explainable learning**, which, through a structure similar to a logical tree, allows for the transparent and comprehensible representation of the causes associated with each type of fault in industrial machinery (Ghasemkhani et al., 2023). This representation not only provides clear justifications for the predictions but also enhances user confidence in the system and facilitates decision-making, enabling end users–such as maintenance technicians—to interpret results more intuitively and act with greater certainty. However, according to the findings, this approach currently requires deep knowledge of industrial machinery operation in order to properly construct and interpret these explanatory structures (Ghasemkhani et al., 2023).

The third key aspect corresponds to the awareness of the existence of **cyberattack vulnerabilities** in models and sensors, which represents a serious threat to their operation and to maintenance activities in general (Tran et al., 2021; Ali et al., 2023; Tran et al., 2023). In interconnected environments typical of Industry 4.0, there are latent risks of external interventions that compromise data integrity–and, consequently, all subsequent processes. For this reason, practices such as false data injection (FDI) (Tran et al., 2023) and adversarial attacks like FGSM and ZOO (Ali et al., 2023) have been proposed to assess and enhance model robustness by distinguishing between real and manipulated data. This reduces the probability of false positives and negatives, preventing erroneous decisions that could affect the correct execution of predictive maintenance based on anomaly analysis and fault prediction classifications.

**Figure 3:**
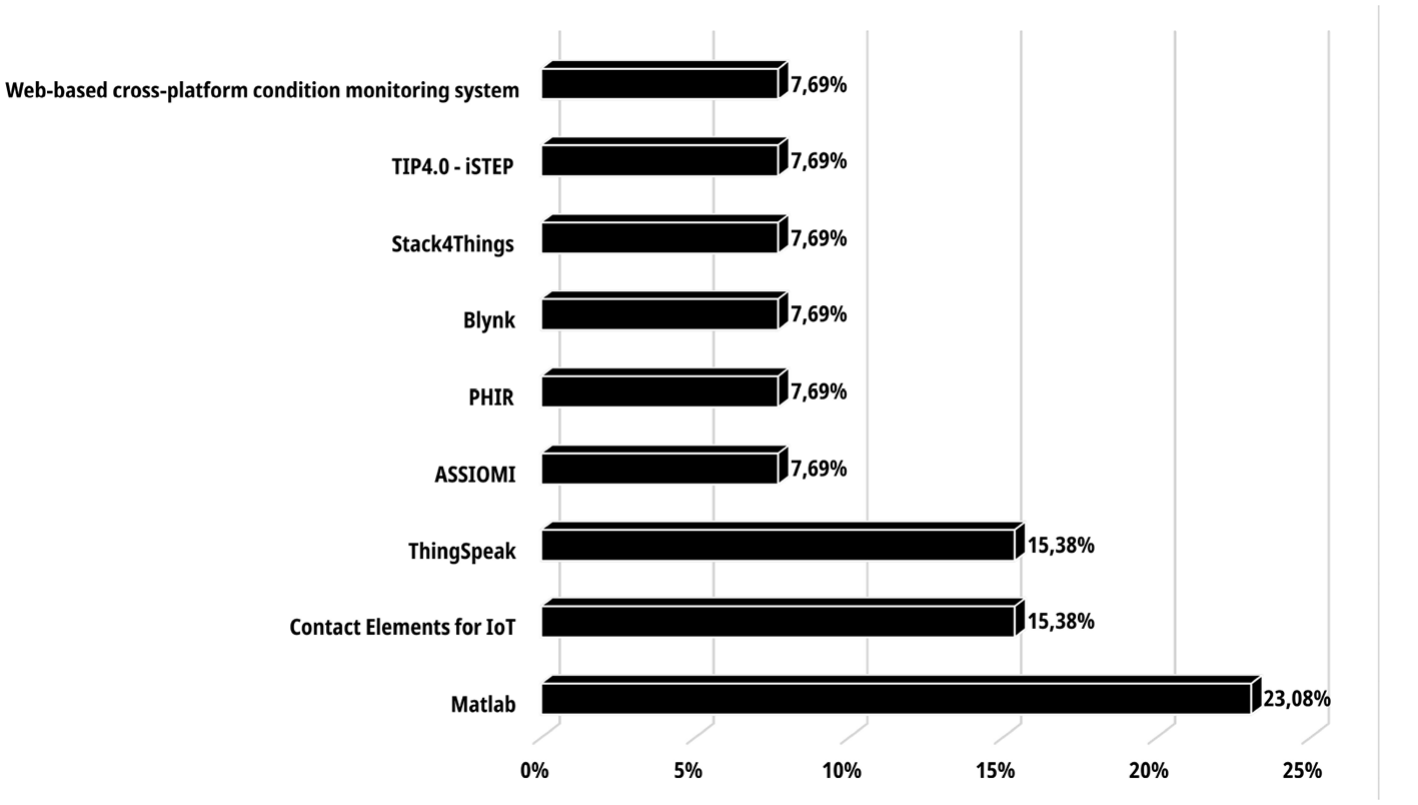
Distribution of software and IoT platform usage.

Regarding software and IoT platforms, Figure 3 presents various technologies that enable the implementation of artificial intelligence algorithms for fault prediction and classification in industrial environments. It can be observed that Matlab was the most widely used software in the literature, due to its excellent integration with tools such as Simulink and its Machine Learning Toolbox, which in turn includes applications like Classification Learner and Regression Learner.

These tools greatly facilitate the integration of Artificial Intelligence algorithms into maintenance activities within the manufacturing industry (Lazzaro et al., 2024; Kazemi et al., 2021; Thoppil et al., 2022). Matlab has even been integrated into platforms such as ThingSpeak and the “Web-based cross-platform condition monitoring system” developed by Xiao et al. (2023), demonstrating how influential Matlab has been in engineering-related activities—particularly in processes involving anomaly analysis and fault prediction and/or classification. Meanwhile, Blynk constitutes a viable option thanks to its use of fixed rules, which allow for the generation of alerts when certain operating parameters exceed their thresholds; it is also simpler to use. Finally, multiple platforms were found that offer a wide range of functionalities that greatly facilitate not only the implementation and deployment of Artificial Intelligence algorithms but also the entire workflow of data management, communication, monitoring, and visualization of industrial assets.

On the other hand, Table 7 presents the **input variables** identified in the review. These represent a fundamental aspect of tasks related to predictive maintenance, particularly in anomaly analysis and fault prediction and classification, since they directly influence the quality of the data used to train artificial intelligence algorithms and, consequently, their performance. It is noteworthy that the vibration variable was the most frequently used throughout the systematic literature review. This is because a large portion of the studies focused on diagnosing the health status of bearings, and this variable best represents their degradation.

**Table 7:** Input variables used in the reviewed studies.

**Input variables used**	**Authors**
Vibration	Ragnoli et al., 2024; Zhao et al., 2022; Magadán et al., 2023; Niu et al., 2023; Maguluri et al., 2024; Liu et al., 2024; Thoppil et al., 2022; Yan et al., 2024; Park et al., 2022; Tran et al., 2021; Chen et al., 2023; Liu et al., 2022; Yousuf et al., 2024; Kizito et al., 2021; Akyaz and Engın, 2024; De Vita et al., 2020; Cao et al., 2024; Li and Zhao, 2022; Zhang et al., 2021; Tran et al., 2023; Han et al., 2021; Mao et al., 2023
Current	Kazemi et al., 2021; Verma et al., 2022; Tran et al., 2021; Yousuf et al., 2024; Akyaz and Engın, 2024; De Vita et al., 2020; Jiang et al., 2024; Tasci et al., 2023; Zhang et al., 2021; Raja et al., 2022
Temperature	Maguluri et al., 2024; Ghasemkhani et al., 2023; Yousuf et al., 2024; Kizito et al., 2021; Akyaz and Engın, 2024; De Vita et al., 2020; Tasci et al., 2023
Speed	Ghasemkhani et al., 2023; Xiao et al., 2023; Yousuf et al., 2024; Jiang et al., 2024; Tasci et al., 2023; Zhang et al., 2021
Voltage	Kazemi et al., 2021; Tran et al., 2021; Yousuf et al., 2024; Zhang et al., 2021; Xiao et al., 2023
Force	Lazzaro et al., 2024; Yotov and Aleksieva-Petrova, 2024; Xiao et al., 2023
Acceleration	Ragnoli et al., 2024; Liu et al., 2024
Pressure	Maguluri et al., 2024; Tasci et al., 2023
Acoustics	Maguluri et al., 2024; De Vita et al., 2020
Vision	Maguluri et al., 2024
Torque	Ghasemkhani et al., 2023
Wear	Ghasemkhani et al., 2023
Distance Proximity	De Vita et al., 2020
Shaft Position	Jiang et al., 2024
Dissolved Gases	Ali et al., 2023
Weight	Tasci et al., 2023
Cyberattack Signals	Tran et al., 2021

Table 8 presents the evaluation metrics for Artificial Intelligence algorithms used in the literature. The use of **evaluation metrics** is essential for AI algorithms applied to anomaly analysis and fault classification and prediction, as these metrics allow for the objective measurement of model performance and the comparison of their effectiveness across different alternatives.

**Table 8:** Evaluation metrics and study authors.

**Evaluation metrics**	**Authors**
Accuracy	Ragnoli et al., 2024; Lazzaro et al., 2024; Zhao et al., 2022; Niu et al., 2023; Maguluri et al., 2024; Kazemi et al., 2021; Liu et al., 2024; Yan et al., 2024; Park et al., 2022; Ghasemkhani et al., 2023; Yotov and Aleksieva-Petrova, 2024; Verma et al., 2022; Tran et al., 2021; Chen et al., 2023; Liu et al., 2022; Yousuf et al., 2024; Kizito et al., 2021; Akyaz and Engın, 2024; Jiang et al., 2024; Ali et al., 2023; Zhang et al., 2021; Tran et al., 2023; Han et al., 2021; Raja et al., 2022
F1-Score	Maguluri et al., 2024; Yan et al., 2024; Park et al., 2022; Ghasemkhani et al., 2023; Kizito et al., 2021; De Vita et al., 2020; Jiang et al., 2024
AUC-ROC	Maguluri et al., 2024; Tran et al., 2021; Jiang et al., 2024; Tran et al., 2023
Precision	Park et al., 2022; Ghasemkhani et al., 2023; Kizito et al., 2021; De Vita et al., 2020
Recall	Park et al., 2022; Ghasemkhani et al., 2023; Kizito et al., 2021; De Vita et al., 2020
Training Time	Niu et al., 2023; Yan et al., 2024
Lead Time	Maguluri et al., 2024; Kizito et al., 2021
Standard Deviation	Park et al., 2022; Li and Zhao, 2022
MSE	Xiao et al., 2023; Kizito et al., 2021
MAPE	Xiao et al., 2023; Li and Zhao, 2022
Coefficient of Determination (R2^)	Xiao et al., 2023; Kizito et al., 2021
Prediction Speed	Lazzaro et al., 2024
Prediction Error	Magadán et al., 2023
RMSE	Thoppil et al., 2022
MAE	Kizito et al., 2021
RMS	Cao et al., 2024
SMAPE	Cao et al., 2024
Score	Cao et al., 2024
Response Time	Jiang et al., 2024
Computational Time	Tran et al., 2023

Finally, Supplementary Table S1 presents a synthesis of the characteristics of each study, including parameters such as the title, authors, dataset size, variables used, algorithms compared with the proposed method, platforms employed, and the evaluation criteria or metrics applied.

### 4.1 Practical Implications

In terms of practical implications, it is recommended that industrial environments where data behavior fluctuates across domains consider using knowledge transfer techniques, as these can be very effective in reducing the impact of such variations (Liu et al., 2024; Mao et al., 2023; Zhao et al., 2022; Cao et al., 2024). It is also crucial to highlight their usefulness in situations where the model must operate under different working conditions or production environments, reducing the number of models needed for a single manufacturing industry (Liu et al., 2024; Mao et al., 2023; Zhao et al., 2022; Cao et al., 2024). In cases with limited computational resources, such as embedded boards used in Edge Computing (Lazzaro et al., 2024; Ali et al., 2023; Xiao et al., 2023; De Vita et al., 2020; Jiang et al., 2024; Cao et al., 2024), or on servers with restricted processing capacity, using Machine Learning-based algorithms, like Support Vector Machines (SVMs) is recommended, as they have proven highly effective in predictive maintenance tasks and require fewer computational resources than neural network-based options (Akyaz and Engın, 2024; Kazemi et al., 2021; Yan et al., 2024; Thoppil et al., 2022; Zhang et al., 2021). In this context, another highly viable alternative is the use of knowledge distillation techniques, which enable complex models, such as neural networks, to transfer their learned knowledge to simpler models that combine robustness, efficiency, and computational lightness (Cao et al., 2024).

In situations where sufficient computational resources are available, it is appropriate to use neural networks to leverage their ability to model complex relationships and process large amounts of data. However, they can also be employed in cases with processing limitations, provided they are combined with knowledge transfer techniques, especially knowledge distillation (Cao et al., 2024). When response time after detecting an anomaly or predicting/classifying a fault is critical, Edge Computing offers a highly effective strategy, as immediate processing on the embedded board prevents delays caused by data transmission (Lazzaro et al., 2024; Ali et al., 2023; Xiao et al., 2023; De Vita et al., 2020; Jiang et al., 2024; Cao et al., 2024). Currently, using Machine Learning algorithms, or a combination of knowledge distillation and neural networks, is a practical approach to achieving faster, satisfactory results and enabling quicker responses to detected events (Akyaz and Engın, 2024; Cao et al., 2024).

**Figure 4:**
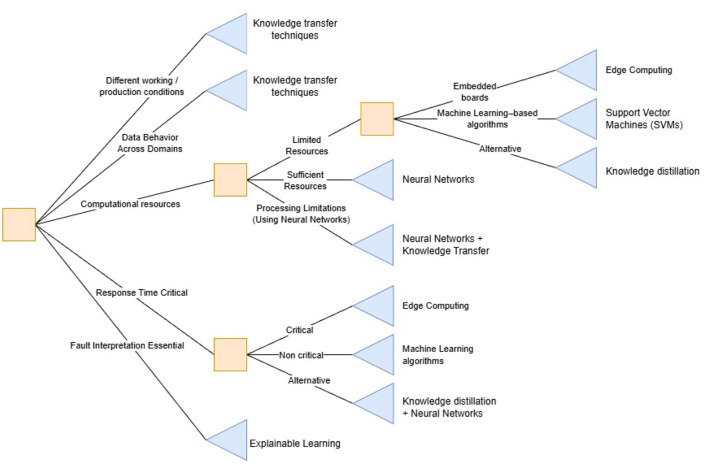
Decision tree on practical implications.

In situations where fault interpretation is essential, the use of explainable learning is deemed appropriate because it opens the “black box” that Artificial Intelligence algorithms often represent, clarifying the models' outputs (Ghasemkhani et al., 2023). Its significance lies in enabling easier analysis and understanding of the detected faults, saving time and resources when interpreting results, and supporting more accurate and faster decision-making. In an industrial context, this can be the difference between success and failure in preventing adverse effects (Ghasemkhani et al., 2023). Figure 4 presents a decision tree with the practical implications of algorithms and technologies for PdM.

## 5 Conclusions

This study conducted a systematic literature review using the method proposed by Kitchenham et al. (2009) to identify artificial intelligence algorithms that enable anomaly analysis and fault prediction-classification, as well as software and IoT platforms that facilitate their implementation within a predictive maintenance context in the manufacturing industry.

The SLR, based on the analyzed studies, concludes that the main AI algorithms for PdM can be categorized into: i) Machine Learning-based methods; ii) Neural Network-based methods; and iii) Knowledge Transfer-based methods. These algorithms demonstrate good results and can be combined. It is also important to highlight the identification of technologies such as Edge Computing and Explainable Learning, along with the increasing need to address cybersecurity. Regarding software and IoT platforms, nine technologies were identified that support the optimization of maintenance operations in the manufacturing industry, offering multiple functions such as monitoring, connectivity, communication, analysis, and visualization. However, the review also reveals that most primary studies provide only limited or generic information about the communication standards supported by these platforms, and only a few explicitly mention protocols such as MQTT, while omitting others, such as OPC-UA. This lack of detailed reporting on interoperability hampers a precise assessment of industrial scalability and should be addressed more systematically in future PdM platform evaluations.

It is advisable that, in most contexts, a data preprocessing and feature processing method be integrated, given the significant benefits these have demonstrated in various studies (Park et al., 2022; Liu et al., 2022; Han et al., 2021; Verma et al., 2022; Raja et al., 2022; Yotov and Aleksieva-Petrova, 2024; Maguluri et al., 2024). Additionally it is desirable to adopt measures aimed at reducing vulnerabilities to cyberattacks, to which both models and sensors may be exposed due to the inherently interconnected nature of Industry 4.0 Tran et al. (2021); Ali et al. (2023); Tran et al. (2023). This would help safeguard maintenance operations against potential impacts from such threats (Tran et al., 2021; Ali et al., 2023; Tran et al., 2023).

In addition to the methodological limitations of this review, it is important to recognize several practical barriers that limit the large-scale industrial adoption of AI-based PdM solutions (Anupama et al., 2022; Abdulrazzq et al., 2024; Hector and Panjanathan, 2024). Empirical studies report that poor data quality–caused by sensor noise, missing values, inconsistent labeling, and heterogeneous data sources–can greatly reduce model reliability and block the development of strong predictive models in real industrial settings (Anupama et al., 2022; Abdulrazzq et al., 2024; Hector and Panjanathan, 2024). Additionally, many studies emphasize that implementing PdM requires significant investments in sensing infrastructure, edge/IIoT devices, data storage, connectivity, and specialized analytics software, which may affect economic viability and slow down return on investment, especially in small and medium-sized enterprises (Florian et al., 2021; Zhu et al., 2025; Zonta et al., 2020; Carvalho et al., 2019). These data-related and integration challenges show that, beyond algorithm improvements, successful industrial adoption of PdM needs coordinated efforts in data governance, system compatibility, and cost-effective deployment strategies that explicitly address investment, operating costs, and value creation (Hector and Panjanathan, 2024; Florian et al., 2021; Zhu et al., 2025; Emmanuel, 2025).

For future work, it is recommended to address the significant gap between the number of studies on AI algorithms and those on software and IoT platforms by conducting empirical research to compare the performance and suitability of these platforms across various industrial sectors. Additionally, fostering collaboration between AI algorithm developers and stakeholders involved in designing and implementing platforms can ensure that progress in both areas aligns and mutually supports each other. To enhance explainable learning, future research should aim to develop tools, algorithms, or strategies that promote its adoption without relying solely on expert knowledge. Finally, given the limited focus on cybersecurity risks in the context of predictive maintenance, it is crucial to investigate methods that improve model resilience against malicious attacks and to implement effective cybersecurity practices in interconnected environments.

## Data Availability

The original contributions presented in the study are included in the article/Supplementary material, further inquiries can be directed to the corresponding author.
