# Supplementary material for: AI algorithms and IoT platforms for anomaly and failure prediction in industrial machinery—systematic review
Source: Front Artif Intell. 2026 Mar 26;9:1799522. doi: 10.3389/frai.2026.1799522 (PMC13061873; doi:10.3389/frai.2026.1799522)
Supplement: Supplementary file 1 [file Supplementary_file_1.pdf]

Supplementary Material

TABLES

Table S1. Synthesis of the SLR articles

Authors	Dataset	Variables	Comparison Algorithms	Proposed Method	Platforms	Evaluation Criteria
Ragnoli *et al.*Ragnoli et al. (2024)	Total: 3241 samples Training: 2139 Testing: 1102	Vibration, acceleration	KNN, SVM, MLP	Regression Tree–Based – CART	ASSIOMI	Weighted average accuracy: 98.7%

Continued on the next page

Authors	Dataset	Variables	Comparison Algorithms	Proposed Method	Platforms	Evaluation Criteria
Lazzaro *et al.*Lazzaro et al. (2024)	210 samples	Cutting force	Fine Tree, Medium Tree, Coarse Tree, Linear Discriminant, Quadratic Discriminant, Quadratic Discriminant, Linear SVM, Quadratic SVM, Cubic SVM, Fine Gaussian SVM, Medium Gaussian SVM, Coarse Gaussian SVM, Fine KNN, Medium KNN, Coarse KNN, Cosine KNN, Cubic KNN, Weighted KNN, Gaussian Kernel Naïve Bayes, Boosted Trees Ensemble, Bagged Trees Ensemble, Subspace Ensemble, Discriminant Ensemble, Subspace KNN Ensemble, RUS Ensemble, Boosted Trees Ensemble, Narrow NN, Medium NN, Wide NN, Trilayered NN	Bilayered NN	Classification Learner (Matlab)	Accuracy: 98.1% Prediction Speed: 12000 s

Continued on the next page

Authors	Dataset	Variables	Comparison Algorithms	Proposed Method	Platforms	Evaluation Criteria
Zhao *et al.*Zhao et al. (2022)	**CWRU dataset:** Total: 9400 samples **DDS dataset:** Total: 6400 samples Training: 70% Testing: 30%	vibración	CNN, TCA, JDA, DANN, ADDA	Joint Adversarial Domain Adaptation (JADA)	–	Average accuracy with DDS dataset: 99.51% Average accuracy with CWRU dataset: 99.67%
Magadán *et al.*Magadán et al. (2023)	IMS Bearing dataset_1: 984 samples IMS Bearing dataset_2: 4448 samples FEMTO Bearing Dataset: * Bearing1_3: 2376 samples * Bearing1_4: 1429 samples * Bearing1_7: 2260 samples XJTU-SY Bearing Dataset: * Bearing2_5: 339 samples * Bearing3_1: 2538 samples Model trained with 70% and tested 30%	vibration	–	SAE-BiLSTM	–	Average prediction error: 1.33%

Continued on the next page

Authors	Dataset	Variables	Comparison Algorithms	Proposed Method	Platforms	Evaluation Criteria
Niu *et al.*Niu et al. (2023)	CWRU dataset QPZZ-II rotating machinery failure test dataset Method 1: 432 samples for training and 68 for testing. Método 2: 1600 samples for training and 400 for testing.	vibration	OMP, OAL, Homotopy, TPTSR, AE, SAE, CNN, LeNet, ResNet18, LSTM, MLP	SR-DEEP	–	CWRU dataset accuracy: 100% QPZZ-II dataset accuracy: 99.20% CWRU dataset average training time: 10487 s QPZZ-II dataset average training time: 10423.8 s
Maguluri *et al.*Maguluri et al. (2024)	2100 samples 150 test samples	Temperature, vibration, pressure, acoustics, vision	RF	Constrastive Predictive Coding (CPC) - NN	TIP4.0 iSTEP	Accuracy: 95.2% AUC-ROC: 98% F1-Score: 93% Average Lead Time: 4.5 h

Continued on the next page

Authors	Dataset	Variables	Comparison Algorithms	Proposed Method	Platforms	Evaluation Criteria
Kazemi *et al.*Kazemi et al. (2021)	Simulated data of internal faults in three-phase transformers in PSCAD/EMTDC<br>* Total: 2800 samples<br>* Training: 1277 samples<br>* Testing: 450 samples	Three-phase currents, three-phase voltages	Recursive Least Squares	Extended Kalman Filter (EFK) - Support Vector Machine (SVM)	Classification Learner de Matlab	Overall accuracy: 98% - 99%
Liu *et al.*Liu et al. (2024)	CWRU dataset: 8000 samples<br>In-house manufacturing dataset: 9000 samples	Vibration, acceleration	ResNet50 without pretraining, ResNet50 with pretraining but without optimizations	Deep Adaptation Residual Neural Network (DARN)	–	Average accuracy: 99%
Thoppil *et al.*Thoppil et al. (2022)	**Experimental accelerated life test bench with 14 vibration signal datasets:**<br>* Training: 10 datasets.<br>* Validation: 2 datasets.<br>* Testing: 2 datasets.	vibration	–	Neighborhood Component Analysis (NCA) - bayesian optimized SVM	Matlab	RMSE: 206.23

Continued on the next page

Authors	Dataset	Variables	Comparison Algorithms	Proposed Method	Platforms	Evaluation Criteria
Yan *et al.*Yan et al. (2024)	**CWRU dataset**: 570 samples<br>**IMS dataset:**<br>* Bearing 1: training 400, testing 100<br>* Bearing 2: training 400, testing 100<br>* Bearing 3: training 400, testing 100<br>* Bearing 4:training 400, testing 100.	vibration	SVM, KNN, SVDD, D-SVDD, F-SVDD, NSVDD, DE-LMANN, IDS-CNN, SAE-DBN	Dinamically Updated Dual-Boundary Support Vector Data Description (DDSVDD)	–	Average F1Score: 97.03%<br>Accuracy: 96.4%<br>Training time: 3.4 min
Park *et al.*Park et al. (2022)	**IMS dataset:**<br>8624 total samples<br>* 4 datasets from this set: 200, 400, 800 y 8624 total samples.	Vibration	DNN with manual supervision, semi-supervised DNN	Auto-labeling-method: Decision Tree + DNN	–	Accuracy: 99%<br>Precision: 99%<br>Recall: 100%<br>F1-Score: 99%<br>std: 0.3%

Continued on the next page

Authors	Dataset	Variables	Comparison Algorithms	Proposed Method	Platforms	Evaluation Criteria
Ghasemkhani *et al.*Ghasemkhani et al. (2023)	AI4I 2020 Predictive Maintenance Dataset: 10000 samples	Air temperature, process temperature, rotation speed, torque, tool wear (minutes)	K-STAR, DFPAIS, SDFIS, EFNC-Exp, SODA, CatBoost, SmoteNC + CatBoost, ctGAN + CatBoost, SmoteNC + ctGAN + CatBoost, XGBoost, RF, TTML + XGBoost, TTML + RF, TTML + MLP, DT, SVM, KNN, HUS-ML, CML, ANN, LR, KNN+LR, Input + LR, AE + LR, Supervised AE, NN, GB, RUSBoost, Constructing Hyper-Planes, Data-Blind Machine Learning, Bagged Decision Trees	Balanced K-STAR	–	F1-Score: 98.75% Recall: 98.75% Precision: 98.77% Accuracy: 98.75%
Yotov y Aleksieva-PetrovaYotov and Aleksieva-Petrova (2024)	Dataset_1: 140 samples Dataset_2: 320 samples	Force	Random Forest Regression (RFR), Multiple Linear Regression (MLR), Decision Tree Regression (DTR), DNN	Wavelet-CNN	–	Accuracy first_dataset: 92.3% Accuracy second_dataset: 62.4%
Verma *et al.*Verma et al. (2022)	36 million data points from induction motors Training: 70% Testing: 30%	Current	KNN, RF, Fuzzy logic (FL), FSA-I NN, Neuro Fuzzy Logic (NFL)	FSA-II NN	–	Accuracy: 98.0%

Continued on the next page

Authors	Dataset	Variables	Comparison Algorithms	Proposed Method	Platforms	Evaluation Criteria
Xiao *et al.*Xiao et al. (2023)	Dataset 1: 13104 samples<br>- Training: 80%<br>- Testing: 20%<br>Dataset 2: 5760 samples<br>- Training: 80%<br>- Testing: 20%	Speed, force, input voltage, and output voltage	SVR, LSTM, GRNN, BPNN, ARIMA	Volterra Polynomial Basis Function (VPBF)	Edge computing-based unified condition monitoring system	Energy consumption prediction: MSE: 0.0649 MAPE: 1.98% $R\hat{2}$: 91.64% Temperature prediction: MSE: 0.0218 MAPE: 0.38% $R\hat{2}$: 91.81%
Tran *et al.*Tran et al. (2021)	Induction motor Dataset: 3079 samples<br>- Training: 70%<br>- Testing: 30%	Vibration, voltage, current, simulated cyberattack signals	XGBoost, DT	Random Forest (RF)	–	Accuracy: 99.03% ROC-AUC: 100%
Chen *et al.*Chen et al. (2023)	**Gearbox dynamics simulator dataset:**<br>* 200 training samples,<br>* 100 testing samples<br>**CWRU Bearing Dataset**	Vibration	MLP, 1D CNN, DBN, Estandard CapsNet, DP-CapsNet, MI-CapsNet, EDR-CapsNet, DPMI-CapsNet, SVM-MLP-Logistic Regression, Continuous Wavelet Transform-ResNet-ELM, VMD-CNN, STFT-MobileNetV2, VMD-Affinity Propagation Clustering, FFT-CapsNet	DPMI-CapsNet	–	Average accuracy: 99%

Continued on the next page

Authors	Dataset	Variables	Comparison Algorithms	Proposed Method	Platforms	Evaluation Criteria
Liu *et al.*Liu et al. (2022)	Drivetrain Diagnostics Simulator (DDS) de Spectra Quest, EE.UU: 8465 samples	Vibration	parametric features + DT, parametric features + BP-NN, parametric features + SVM, parametric features + SCN, stacking features + BP-NN, stacking features + SVM, stacking features + SCN	Stacking features + Decision tree	–	Accuracy: 99.94%
Yousuf *et al.*Yousuf et al. (2024)	Induction motor sensor data	Temperature, vibration, current, voltage, speed ratio	–	–	Blynk	Accuracy: 99%
Kizito *et al.*Kizito et al. (2021)	Motor Degradation Dataset: *** Failure Prediction:** - Training: 36335 observations - Testing: 7255 observations *** RUL Prediction** - Training: 28981 observations - Testing: 5832 observaciones	Vibration, temperature	RF	Stylized LSTM	–	**Failure Prediction Results:** * Accuracy: 93.4% * Precision: 100% * Recall: 83.3% * F1-Score: 90.9% * Tiempo de predicción de la falla: 24 horas antes **RUL results:** * MAE: 0.075 * MSE: 0.008 * R2̂: 92%

Continued on the next page

Authors	Dataset	Variables	Comparison Algorithms	Proposed Method	Platforms	Evaluation Criteria
Akyaz y EnginAkyaz and Engın (2024)	Operational data from a Bulk Continuous Filament (BCF) machine used in artificial yarn production: 2,800 records	Vibration, temperature, current	RF, SVM, SVR, DT, GP	DNN	ThingSpeak MATLAB Regression Learner	Accuracy: 96% Prediction validation score: 86%
De Vita *et al.*De Vita et al. (2020)	Data generated from a real-scale industrial test bench: 20,000 samples; K-fold cross-validation = 10	Acoustic noise, distance/proximity, current, vibration, temperature	Monolithic DNN, SVM	Deep Neural Network (DNN)	Stack4Things	Average Precision: 95% Average Recall: 94% Average F1-Score: 94%
Cao *et al.*Cao et al. (2024)	PHM Challenge 2012 Bearings Dataset: 2560 data points per sample; ABLT-1A Bearings Dataset: 25600 data points per sample	Vibration	Vanilla KD, UMLKD, Self-KD, OKD with TBR loss, OKD with TOR loss, OKD with RKD loss, OKD with RAE and ETFIT	OKD - Vanilla CNN	–	RMS: 0.1002 SMAPE: 57.27% Score: 57.64%

Continued on the next page

Authors	Dataset	Variables	Comparison Algorithms	Proposed Method	Platforms	Evaluation Criteria
Li y ZhaoLi and Zhao (2022)	**PRONOSTIA Rolling Bearings Dataset:** - bearing1_5: 2442 * 2000 (Training y Testing 442) - bearing2_5: 2304 * (Training 1900; Testing 404) - bearing3_2: 1625 samples (Training: 1200; Testing: 425	Vibration	PSO-KELM, WPSO-KELM, CPSO-KELM, DPSO-KELM	WCDPSO-KELM	–	Average MAPE: 6.86% std: 0.0013

Continued on the next page

Authors	Dataset	Variables	Comparison Algorithms	Proposed Method	Platforms	Evaluation Criteria
Jiang *et al.*Jiang et al. (2024)	**PHIR Industrial Robot Dataset Dataset collected from real industrial robots Dataset:**<br>- A: 216000 training samples and 2400000 testing samples, (3.38% anomalies)<br>- Datasete B: 4080000 training samples and 4680000 testing samples (2.97% anomalies)	Current, joint position, actuator speed	PCA, t-SNE, AE, AE-C, LSTM, GRU, Transformer, Transformer-PS	AE-DTW, Transformer-SD	PHIR	**PHIR:**<br>- Accuracy: 81.8%<br>- Response time: 5s<br>**AE-DTW:**<br>- Average F1-SCORE: 73.35%<br>- Average AUC-ROC: 69.95%<br>**Transformer - SD:**<br>- Average F1-SCORE: 67.7%<br>- Average AUC-ROC: 58.8%
Ali *et al.*Ali et al. (2023)	Laboratory data and academic literature on dissolved gases in transformer insulating oil: 448 total samples. Training: 80%; Testing: 20%	**Dissolved gases:** H2, CH4, C2H2, C2H4, C2H6, CO y CO2 en el aceite aislante del transformador	LR, LDA, GNB, SVC, Duval, IEC, Rogers, ANN, 1D-CNN	Gradient tree boosting (GTP)	Contact Elements for IoT	Accuracy training: 99.7%<br>Accuracy test: 89.7%

Continued on the next page

Authors	Dataset	Variables	Comparison Algorithms	Proposed Method	Platforms	Evaluation Criteria
Taşcı *et al.*Tasci et al. (2023)	IoT sensor data from production lines of consumer goods and shutdown data. Training: 70%; Testing: 30%	Weight, speed, temperature, current, vacuum, air pressure	AE-Kmeans-XGB, AE-Kmeans-SVR, AE-Kmeans-MLP	AE-Kmeans-RF	–	Detected 42% of real failures in advance
Zhang *et al.*Zhang et al. (2021)	Experimental sensor data from rotating machinery systems: 4,088 total samples. Divided into: Level 1: 2,045 samples Level 2: 1,026 samples Level 3: 522 samples Level 4: 258 samples s	Vibration, speed, voltage, current	DT, RF, NN	Featured Oriented Support Vector Machine (FO-SVM)	ThingSpeak	Accuracy: 98.2%

Continued on the next page

Authors	Dataset	Variables	Comparison Algorithms	Proposed Method	Platforms	Evaluation Criteria
Tran *et al.*Tran et al. (2023)	Experimental data from induction motors in a laboratory environment: 3,079 samples. Training: 70%; Testing: 30%; Visualization: 615 samples	Vibration	1D-CNN, SVM, KNN, DT, RF	DNN	Contact Elements for IoT	Accuracy: 99.84% AUC-ROC: aprox. 100% Computational Time: 0.038s
Han *et al.*Han et al. (2021)	CWRU Dataset: 480 samples	Vibration	EMD/SVD/FNN, Orthogonal neighborhood preserving embedding (ONPE)/Adaboost-SVM, Adaptive stochastic resonance/AMD/EEMD, CEEMDAN/distance evaluation technique/SVM, EMD/energy entropy of the first six IMFs / least squares support vector machine(LSSVM), CEEMD/Kernel-SVM, Improved VMD/SVD/DBN	LMD-MSDE-AP	–	Classification accuracy: 99.38%

Continued on the next page

Authors	Dataset	Variables	Comparison Algorithms	Proposed Method	Platforms	Evaluation Criteria
Mao *et al.*Mao et al. (2023)	**IEEE PHM Challenge 2012 Bearing Dataset:** divided into: Bearing_2_1, Bearing2_2, Bearing2_4, Bearing2_6 **XJTU-SY Bearing Dataset:** bearing 2_1, bearing 2_2	Vibration	SVR, LSTM, Tensor-LSTM, Deutsch's method, Mao's method, Zhu's method, TCA, KMM, GFK, SA, Sun's method, DANN, Costa´s method	Self-Supervised Deep Domain-Adversarial Regression Adaptation	–	–

Continued on the next page

Authors	Dataset	Variables	Comparison Algorithms	Proposed Method	Platforms	Evaluation Criteria
Raja *et al.*Raja et al. (2022)	- Training: 68,000 samples Testing: 6,800 samples	Current	Signal Spectrum-Based Machine Learning Approach - Course Tree, Signal Spectrum-Based Machine Learning Approach - Gaussian Naïve Bayes, Signal Spectrum-Based Machine Learning Approach - Fine KNN, Signal Spectrum-Based Machine Learning Approach - Narrow NN, Signal Spectrum-Based Machine Learning Approach -Medium NN, Signal Spectrum-Based Machine Learning Approach - Bilayered NN, Signal Spectrum-Based Machine Learning Approach - Trilayered NN	Signal Spectrum-Based Machine Learning Approach - Wide Neural Network	–	Accuracy: 99.6%

REFERENCES

Ragnoli M, Pavone M, Epicoco N, Pola G, De Santis E, Barile G, et al. A Condition and Fault Prevention Monitoring System for Industrial Computer Numerical Control Machinery. *IEEE ACCESS* **12** (2024) 20919–20930. doi:10.1109/ACCESS.2024.3359424. Num Pages: 12 Place: Piscataway Publisher: Ieee-Inst Electrical Electronics Engineers Inc Web of Science ID: WOS:001161991700001.

Lazzaro A, D'Addona DM, Merenda M. A Detailed Study on Algorithms for Predictive Maintenance in Smart Manufacturing: Chip Form Classification Using Edge Machine Learning. *IEEE Open Journal of the Industrial Electronics Society* **5** (2024) 1190–1205. doi:10.1109/OJIES.2024.3484006. Conference Name: IEEE Open Journal of the Industrial Electronics Society.

Zhao X, Shao F, Zhang Y. A Novel Joint Adversarial Domain Adaptation Method for Rotary Machine Fault Diagnosis under Different Working Conditions. *SENSORS* **22** (2022) 9007. doi:10.3390/s22229007. Num Pages: 17 Place: Basel Publisher: MDPI Web of Science ID: WOS:000887614000001.

Magadán L, Suárez F, Granda J, delaCalle F, García D. A Robust Health Prognostics Technique for Failure Diagnosis and the Remaining Useful Lifetime Predictions of Bearings in Electric Motors. *Applied Sciences (Switzerland)* **13** (2023). doi:10.3390/app13042220. Publisher: MDPI.

Niu Y, Deng W, Zhang X, Wang Y, Wang G, Wang Y, et al. A Sparse Learning Method with Regularization Parameter as a Self-Adaptation Strategy for Rolling Bearing Fault Diagnosis. *ELECTRONICS* **12** (2023) 4282. doi:10.3390/electronics12204282. Num Pages: 17 Place: Basel Publisher: MDPI Web of Science ID: WOS:001095282400001.

Maguluri LP, Suganthi D, Dhote GM, Kapila D, Jadhav MM, Neelima S. AI-enhanced predictive maintenance in hybrid roll-to-roll manufacturing integrating multi-sensor data and self-supervised learning. *The International Journal of Advanced Manufacturing Technology* (2024). doi:10.1007/s00170-024-14263-7.

Kazemi Z, Naseri F, Yazdi M, Farjah E. An EKF-SVM machine learning-based approach for fault detection and classification in three-phase power transformers. *IET SCIENCE MEASUREMENT & TECHNOLOGY* **15** (2021) 130–142. doi:10.1049/smt2.12015. Num Pages: 13 Place: Hoboken Publisher: Wiley Web of Science ID: WOS:000606639600001.

Liu S, Ji Z, Zhang Z, Wang Y. An Improved Deep Transfer Learning Method for Rotating Machinery Fault Diagnosis Based on Time Frequency Diagram and Pretraining Model. *IEEE Transactions on Instrumentation and Measurement* **73** (2024) 1–12. doi:10.1109/TIM.2023.3345907. Conference Name: IEEE Transactions on Instrumentation and Measurement.

Thoppil NM, Vasu V, Rao CSP. An Integrated Learning Algorithm for Vibration Feature Selection and Remaining Useful life Estimation of Lathe Spindle Unit. *Journal of Failure Analysis and Prevention* **22** (2022) 1693–1701. doi:10.1007/s11668-022-01463-0.

Yan A, Zhao Y, Lu Z, Pang Y, Jin S, Liu Z, et al. An Online Fault Detection and Remaining Life Prediction Method Based on SVDD for Rolling Bearings. *IEEE Transactions on Instrumentation and Measurement* **73** (2024) 1–12. doi:10.1109/TIM.2024.3378268. Conference Name: IEEE Transactions on Instrumentation and Measurement.

Park S, Ahn GJ, Im DH. Auto Labeling Methods Developed Through Semi-Weakly Supervised Learning in Prognostics and Health Management Applications for Rolling Ball Bearing. *IEEE Sensors Journal* **22** (2022) 16223–16233. doi:10.1109/JSEN.2022.3188310. Conference Name: IEEE Sensors Journal.

Ghasemkhani B, Aktas O, Birant D. Balanced K-Star: An Explainable Machine Learning Method for Internet-of-Things-Enabled Predictive Maintenance in Manufacturing. *Machines* **11** (2023). doi:10.3390/machines11030322. Publisher: MDPI.

Yotov O, Aleksieva-Petrova A. Data-Driven Prediction Model for Analysis of Sensor Data. *Electronics (Switzerland)* **13** (2024). doi:10.3390/electronics13101799. Publisher: Multidisciplinary Digital Publishing Institute (MDPI).

Verma A, Raval P, Rajagopalan N, Khariya V, Sudha R. Development of an AI-based FSA for real-time condition monitoring for industrial machine. *Neural Computing and Applications* **34** (2022) 8597–8615. doi:10.1007/s00521-021-06741-w. Publisher: Springer Science and Business Media Deutschland GmbH.

Xiao H, Hu W, Liu G, Zhou H. Edge computing-based unified condition monitoring system for process manufacturing. *Computers and Industrial Engineering* **177** (2023). doi:10.1016/j.cie.2023.109032. Publisher: Elsevier Ltd.

Tran MQ, Elsisi M, Mahmoud K, Liu MK, Lehtonen M, Darwish MMF. Experimental Setup for Online Fault Diagnosis of Induction Machines via Promising IoT and Machine Learning: Towards Industry 4.0 Empowerment. *IEEE ACCESS* **9** (2021) 115429–115441. doi:10.1109/ACCESS.2021.3105297. Num Pages: 13 Place: Piscataway Publisher: Ieee-Inst Electrical Electronics Engineers Inc Web of Science ID: WOS:000688220400001.

Chen H, Wang Xb, Yang ZX. Fast Robust Capsule Network With Dynamic Pruning and Multiscale Mutual Information Maximization for Compound-Fault Diagnosis. *IEEE/ASME Transactions on Mechatronics* **28** (2023) 838–847. doi:10.1109/TMECH.2022.3214865. Conference Name: IEEE/ASME Transactions on Mechatronics.

Liu Q, Zhang J, Liu J, Yang Z. Feature extraction and classification algorithm, which one is more essential? An experimental study on a specific task of vibration signal diagnosis. *International Journal of Machine Learning and Cybernetics* **13** (2022) 1685–1696. doi:10.1007/s13042-021-01477-4.

Yousuf M, Alsuwian T, Amin A, Fareed S, Hamza M. IoT-based health monitoring and fault detection of industrial AC induction motor for efficient predictive maintenance. *Measurement and Control (United Kingdom)* **57** (2024) 1146–1160. doi:10.1177/00202940241231473. Publisher: SAGE Publications Ltd.

Kizito R, Scruggs P, Li X, Devinney M, Jansen J, Kress R. Long Short-Term Memory Networks for Facility Infrastructure Failure and Remaining Useful Life Prediction. *IEEE Access* **9** (2021) 67585–67594. doi:10.1109/ACCESS.2021.3077192. Conference Name: IEEE Access.

Akyaz T, Engın D. Machine Learning-Based Predictive Maintenance System for Artificial Yarn Machines. *IEEE Access* **12** (2024) 125446–125461. doi:10.1109/ACCESS.2024.3454548. Conference Name: IEEE Access.

De Vita F, Bruneo D, Das SK. On the use of a full stack hardware/software infrastructure for sensor data fusion and fault prediction in industry 4.0. *PATTERN RECOGNITION LETTERS* **138** (2020) 30–37. doi:10.1016/j.patrec.2020.06.028. Num Pages: 8 Place: Amsterdam Publisher: Elsevier Web of Science ID: WOS:000579804900005.

Cao Y, Ni Q, Jia M, Zhao X, Yan X. Online Knowledge Distillation for Machine Health Prognosis Considering Edge Deployment. *IEEE Internet of Things Journal* **11** (2024) 27828–27839. doi:10.1109/JIOT.2024.3404112. Conference Name: IEEE Internet of Things Journal.

Li X, Zhao H. Performance Prediction of Rolling Bearing Using EEMD and WCDPSO-KELM Methods. *APPLIED SCIENCES-BASEL* **12** (2022) 4676. doi:10.3390/app12094676. Num Pages: 19 Place: Basel Publisher: MDPI Web of Science ID: WOS:000795236600001.

Jiang F, Hu C, Liu C, Wang R, Zhu J, Chen S, et al. PHIR: A Platform Solution of Data-Driven Health Monitoring for Industrial Robots. *ELECTRONICS* **13** (2024) 834. doi:10.3390/electronics13050834. Num Pages: 17 Place: Basel Publisher: MDPI Web of Science ID: WOS:001182754600001.

Ali MN, Amer M, Elsisi M. Reliable IoT Paradigm With Ensemble Machine Learning for Faults Diagnosis of Power Transformers Considering Adversarial Attacks. *IEEE Transactions on Instrumentation and Measurement* **72** (2023) 1–13. doi:10.1109/TIM.2023.3300444. Conference Name: IEEE Transactions on Instrumentation and Measurement.

Tasci B, Omar A, Ayvaz S. Remaining useful lifetime prediction for predictive maintenance in manufacturing. *COMPUTERS & INDUSTRIAL ENGINEERING* **184** (2023) 109566. doi:10.1016/j.cie.2023.109566. Num Pages: 18 Place: Oxford Publisher: Pergamon-Elsevier Science Ltd Web of Science ID: WOS:001070887700001.

Zhang X, Rane KP, Kakaravada I, Shabaz M. Research on vibration monitoring and fault diagnosis of rotating machinery based on internet of things technology. *NONLINEAR ENGINEERING - MODELING*

AND APPLICATION **10** (2021) 245–254. doi:10.1515/nleng-2021-0019. Num Pages: 10 Place: Berlin Publisher: Walter De Gruyter Gmbh Web of Science ID: WOS:000714688100001.

Tran MQ, Amer M, Dababat A, Abdelaziz AY, Dai HJ, Liu MK, et al. Robust fault recognition and correction scheme for induction motors using an effective IoT with deep learning approach. *MEASUREMENT* **207** (2023) 112398. doi:10.1016/j.measurement.2022.112398. Num Pages: 12 Place: Oxford Publisher: Elsevier Sci Ltd Web of Science ID: WOS:000917086600001.

Han M, Wu Y, Wang Y, Liu W. Roller bearing fault diagnosis based on LMD and multi-scale symbolic dynamic information entropy. *Journal of Mechanical Science and Technology* **35** (2021) 1993–2005. doi:10.1007/s12206-021-0417-3.

Mao W, Chen J, Liu J, Liang X. Self-Supervised Deep Domain-Adversarial Regression Adaptation for Online Remaining Useful Life Prediction of Rolling Bearing Under Unknown Working Condition. *IEEE Transactions on Industrial Informatics* **19** (2023) 1227–1237. doi:10.1109/TII.2022.3172704. Conference Name: IEEE Transactions on Industrial Informatics.

Raja H, Kudelina K, Asad B, Vaimann T, Kallaste A, Rassõlkin A, et al. Signal Spectrum-Based Machine Learning Approach for Fault Prediction and Maintenance of Electrical Machines. *Energies* **15** (2022). doi:10.3390/en15249507. Publisher: MDPI.
